# Sustainable Valorization of Pb(II)-Loaded *Inga edulis* Biomass: From Adsorption to Oxygen Evolution Electrocatalysis

**DOI:** 10.1021/acsomega.6c05335

**Published:** 2026-09-09

**Authors:** Lucas dos S. Lima, Erica P. Fernandes, Andrea Novelli, Angel Tomé de L. Silva, Luiz Pereira da Costa, Marcos V. Quirino dos Santos, Jefferson A. Freitas, Eliana M. Sussuchi

**Affiliations:** † Research Group in Electrochemical Sensors and Nano(Materials) (SEnM), Laboratory of Corrosion and Nanotechnology (LCNT), Graduate Program in Chemistry, Department of Chemistry, 74391 Federal University of Sergipe, Av. Marcelo Deda Chagas, 304, Rosa Elze, São Cristóvão, Sergipe 49107-230, Brazil; ‡ Aquatic Ecosystems Study Group (GEEA), Department of Environmental Engineering, Federal University of Sergipe, Av. Marcelo Deda Chagas, 304, Rosa Elze, São Cristóvão, Sergipe 49107-230, Brazil; § Laboratory of Nanotechnology and Functional Materials (LANanMF), Department of Chemistry, Federal University of Sergipe, Av. Marcelo Deda Chagas, 304, Rosa Elze, São Cristóvão, Sergipe 49107-230, Brazil; ∥ Environmental Technology Laboratory (LATAM), Department of Environmental Engineering, Federal University of Sergipe, Av. Marcelo Deda Chagas, 304, Rosa Elze, São Cristóvão, Sergipe 49107-230, Brazil

## Abstract

This work reports on the removal of Pb­(II) ions from aqueous solution using *Inga edulis* biomass modified with sulfuric acid (BMIGA), evaluating its performance in a fixed-bed system and potential ecotoxicological effects after the adsorption process. Physicochemical characterization of the material showed that the modification increased the amount of active functional groups, consequently enhancing interaction with metal ions. Batch adsorption tests indicated a strong influence of pH on the removal efficiency, with an optimal pH close to 6.0. The adsorption capacity increased with decreasing particle size and adequate adsorbent dosage. The Thomas and Yoon-Nelson models provided the best fits the experimental data obtained in fixed-bed adsorption experiments, with coefficients of determination close to 0.99 indicating high performance of the system. After the adsorption process, the material saturated with Pb­(II) was applied as an electrocatalyst for the oxygen evolution reaction (OER), exhibiting reduced overpotential and a significant improvement in current density. In ecotoxicological tests with *Daphnia similis*, an EC_50.48h_ value of 13.6 μg L^–1^ indicated the toxicity of Pb­(II), while no acute effects were observed for the post-adsorption supernatants, demonstrating the efficiency of BMIGA in completely removing the metal. It can be concluded that BMIGA represents a sustainable and effective alternative material for the treatment of lead-containing effluents, exhibiting a maximum adsorption capacity of 222.14 mg g^–1^. In addition to its excellent adsorptive performance, BMIGA also shows potential for reuse as an electrocatalyst and presents favorable environmental safety characteristics.

## Graphical abstract

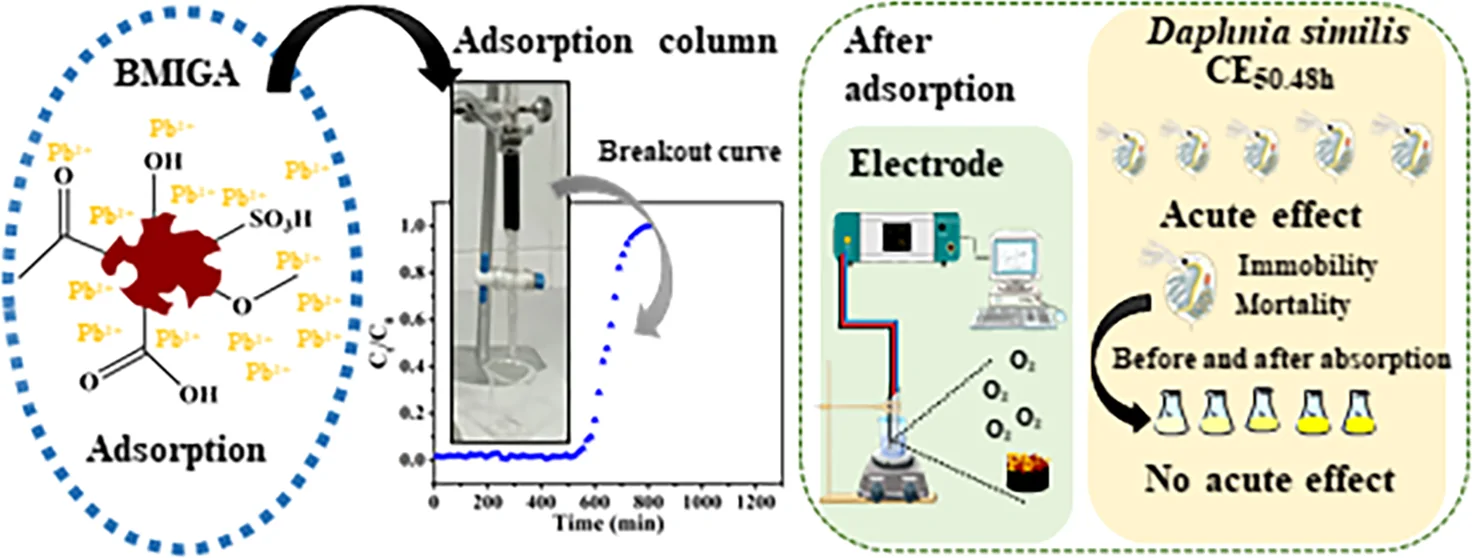

## 1 Introduction

Increasing global urbanization and industrialization is associated with discharges of considerable volumes of untreated effluents containing heavy metals into rivers and oceans, leading to the contamination of aquatic ecosystems.^1^ The lead­(II) ion is one of the most significant toxic metal species, due to its capacity to bioaccumulate and cause adverse effects including interference in biochemical processes, damage to the vital organs of humans and animals, and serious diseases.^2,3^

A wide range of Pb­(II) concentrations have been reported in aquatic environments and sediments in many countries, such as 313.40 mg L^–1^ (Iran), 0.01 mg L^–1^ (Romania), 0.01 mg L^–1^ (Indonesia), 11.80 mg kg^–1^ (Egypt), 314.00 mg kg^–1^ (Italy), and 30.20 mg kg^–1^ (Brazil).^4–8^ In Brazil, the National Environmental Council (CONAMA) has established maximum permissible limits for Pb­(II) of 0.01 mg L^–1^ in drinking water and 35.00 mg kg^–1^ in sediments.^9^ The World Health Organization (WHO) recommends a Pb­(II) limit of 0.01 mg L^–1^ in water intended for human consumption.^10^

The occurrence of Pb­(II) in aquatic ecosystems and sediments is strongly associated with mining activities, industrial processes, and poor waste disposal practices.^11^ Under alkaline conditions, lead tends to precipitate as oxides or hydroxides, while in acidic media it remains predominantly in the ionic Pb­(II) form, which increases its bioavailability and, consequently, the risks to aquatic biota.^12^ Therefore, it is essential to be able to monitor and remove this contaminant, to ensure water quality and environmental preservation.

The aquatic biota comprises a diverse range of microorganisms, including microcrustacean species, such as *Daphnia similis*. This species is recognized as a highly sensitive natural bioindicator of changes in the chemical composition of the environment, particularly the presence of metals and organic compounds.^13^ Several studies have employed *D. similis* for the early detection of toxic metals at trace concentrations in aquatic environments.^14,15^ An acute effect of the presence of Pb­(II) ions has been observed, with an effective concentration (EC_50_) value of 0.34 mg L^–1^.^16^ Hence, it is important that contamination affecting the aquatic biota should be rapidly identified, with the implementation of strategies to mitigate the environmental impacts resulting from this type of pollutant.

Several techniques have been employed to reduce Pb­(II) concentrations in aquatic environments, including chemical precipitation, sand filtration, ion exchange, membrane-based processes, bioremediation using Ganoderma species in mycoremediation bioreactors, and adsorption.^17–20^ Among these approaches, adsorption has emerged as one of the most attractive treatment technologies owing to its cost-effectiveness, operational simplicity, high removal efficiency, and potential for adsorbent regeneration.^21^

Recent advances in adsorption science have resulted in the development of a broad range of adsorbent materials for the remediation of metal ions and organic pollutants in aqueous systems. These materials include rice husk-derived silica, metal–organic frameworks (MOFs), bio-based adsorbents, polymeric composites, nanostructured materials, and chitosan-based hybrid systems.^11,22–25^ Such materials have shown excellent performance in the treatment of industrial effluents generated by sectors including mining, electroplating, battery manufacturing, textile processing, and chemical production.^18,26–28^ Recent studies have emphasized the growing demand for sustainable and multifunctional adsorbents with enhanced adsorption capacity, selectivity, reusability, and environmental compatibility.^29^

Despite these advances, important challenges remain. Many high-performance adsorbents require complex synthesis routes, exhibit limited stability under practical operating conditions, or involve relatively high production costs, thereby restricting their large-scale application.^30–32^ Furthermore, the development of environmentally benign adsorbents capable of efficiently removing toxic heavy metals while maintaining structural integrity and regeneration capacity remains an important research challenge.^33,34^ In this context, the present study contributes to the development of efficient and sustainable adsorbent materials for Pb­(II) removal from aqueous media. This research is driven by the growing need for economically viable technologies to reduce lead contamination, which continues to pose serious environmental and public health risks.

Recent years have seen increasing attention given to the use of plant-derived carbonaceous materials to produce adsorbents, since these are renewable and environmentally sustainable resources, with properties that vary depending on the species used.^35,36^ Recent research has investigated the potential of *Inga edulis* as a precursor to produce adsorbent materials, mainly due to its high lignocellulosic content.^37^ This species belongs to the Fabaceae family and is native to the Brazilian Amazon region, where it is commonly known as “ingá”, “ingá-de-metro”, “ingá-feijão”, and “ingá-doce”.^38^ The fruit peel contains approximately 70.00% insoluble fiber, primarily composed of cellulose, hemicellulose, and lignin, highlighting its suitability as a renewable precursor for the production of adsorbent materials.

Oxygenated and nitrogen-containing functional groups present in lignocellulosic compounds play important roles in interactions with metal ions and other pollutants, contributing to reducing their concentrations in aquatic environments.^39^ In addition to these functional groups, materials with high carbon content often exhibit high porosity and high surface areas, which are characteristics that can significantly enhance contaminant removal.^40,41^ Recent studies have investigated the use of physical and chemical treatments to improve the adsorptive capacities of carbonaceous materials. For example, modification with an acidic agent, such as sulfuric acid, can result in specific structural changes that enhance the adsorption efficiency for metal ions.^42,43^

In the present study, *I. edulis* peel was activated with sulfuric acid and evaluated for Pb­(II) removal through batch and continuous-flow adsorption systems. Batch experiments were performed to investigate the adsorption behavior by means of kinetic, equilibrium, thermodynamic, selectivity, and regeneration studies, while fixed-bed column experiments were conducted to assess the applicability of the material under conditions more representative of industrial wastewater treatment. Following adsorption, ecotoxicological assays were performed to verify the reduction in toxicity, and the Pb-loaded adsorbent was subsequently evaluated as an electrocatalyst for the oxygen evolution reaction (OER).

The novelty of this work lies in integrating continuous-flow Pb­(II) remediation with ecotoxicological validation and the valorization of the spent adsorbent as an electrocatalytic material, thereby combining wastewater treatment, environmental safety, and resource recovery within a circular economy framework. Previous work has provided a comprehensive characterization of the lignocellulosic structure of *I. edulis*, demonstrating its potential as a precursor for adsorbent production and its application in Cr­(VI) adsorption.^37^ However, that study was limited to batch adsorption and did not investigate fixed-bed column operation or the practical implementation of the material under continuous-flow conditions. Therefore, the present work substantially expands the previous research by demonstrating the applicability of the adsorbent in dynamic systems, validating the environmental safety of the treated effluent through ecotoxicological assays, and introducing a sustainable post-adsorption valorization strategy via oxygen evolution electrocatalysis.

## 2 Materials and Methods

### 2.1 Chemicals

Lead acetate (Pb­(CH_3_COO)_2_), calcium chloride (CaCl_2_), potassium nitrate (KNO_3_), sodium chloride (NaCl), and sodium acetate (NaCH_3_COO) were purchased from Dinâmica (purity ≥99.00%). Sulfuric acid (H_2_SO_4_) was obtained from CRQ (purity of 98.10%). Potassium bromide (KBr) and sodium sulfate (Na_2_SO_4_) were supplied by Merck (purity ≥99.00%). Nitric acid (HNO_3_, 65.00%) and hydrochloric acid (HCl, 37.00%) were acquired from Neon. Solutions containing Pb­(II) ions were prepared with ultrapure water produced by a Milli-Q purification system (Merck Millipore).

### 2.2 Adsorbent Preparation

The adsorbent material was obtained from the peels of *Inga* sp. fruits, which were collected in the municipality of Itacoatiara (Amazonas state, Brazil) and were subsequently modified by the Functional Materials Research Group at the Federal University of Sergipe (LANanMF-SE). The stages of collection, preparation, and activation of the material with sulfuric acid were performed as described in a previous study by the same research group.^37^ The unmodified material was labeled BMIG and the acid-activated material was labeled BMIGA.

### 2.3 Characterizations

The morphological characteristics and elemental composition of the materials were examined by scanning electron microscopy (SEM) coupled with energy-dispersive X-ray spectroscopy (EDS) using a VEGA 3 LMS microscope (TESCAN). Prior to analysis, the samples were mounted on carbon tape attached to a copper specimen holder and coated with a thin gold layer by sputtering. Surface elemental composition was further investigated by X-ray photoelectron spectroscopy (XPS) using a K-Alpha spectrometer (Thermo Scientific). Survey and high-resolution spectra were collected over an energy range of 0–1200 eV with energy resolutions of 1.0 and 0.1 eV, respectively. High-resolution spectra were acquired for the C 1s, O 1s, and Pb 4f regions, and the resulting data were processed using Thermo Avantage software.

### 2.4 Adsorption Experiments

The Pb­(II) ion adsorption experiments were performed in batch mode, in triplicate, with lead acetate (Pb­(CH_3_COO)_2_) as the precursor salt. The residual Pb­(II) concentrations were quantified by UV–vis spectrophotometry (Cary 100, Varian) and flame atomic absorption spectrometry (AAS) (AA-700, Shimadzu). Information concerning the calibration curve is provided in Section S1 of the Supporting Material. Batch adsorption experiments were conducted in 250.00 mL Erlenmeyer flasks containing 20.00 mL of Pb­(II) solution and the adsorbent materials (BMIG or BMIGA), under continuous agitation at 150 rpm. The effects of pH, particle size, and adsorbent dosage were optimized at 25.0 °C using a contact time of 24 h.

The effect of pH on Pb­(II) adsorption was investigated over the pH range of 2.0–11.0 using 200.00 mg L^–1^ Pb­(II) solutions and 20.00 mg of adsorbent. The pH was adjusted with 0.10 mol L^–1^ HCl or NaOH solutions. The influence of particle size was evaluated using 20.00 mg of adsorbent with particle sizes ranging from 0.500 to 0.075 mm in 200.00 mg L^–1^ Pb­(II) solutions at pH 6.0. The effect of adsorbent dosage was examined by varying the adsorbent concentration from 0.25 to 3.00 g L^–1^ while maintaining the Pb­(II) concentration at 200.00 mg L^–1^ and the solution pH at 6.0. The performances of the materials for adsorption of Pb­(II) ions were calculated using eqs ([Disp-formula eq1]–[Disp-formula eq3]).

1 $$r\mathrm{emoval}\;\mathrm{efficiency}=\frac{{(C}_{i}-C_{e})}{C_{i}}\times 100\%$$

2 $$q_{t}=\frac{{(C}_{i}-C_{t})}{m}\times V$$

3 $$q_{e}=\frac{{(C}_{i}-C_{e})}{m}\times V$$

where (*C*_i_), (*C*_t_), and (*C*_e_) represent the Pb­(II) concentrations (mg L^–1^) at the initial time, at time (*t*), and at equilibrium, respectively. Likewise, (*q*_t_) and (*q*_e_) denote the adsorption capacities (mg g^–1^) at time (*t*) and at equilibrium, respectively. The parameter *V* is the solution volume (L), while *m* is the mass of adsorbent used (g).

### 2.5 Adsorption Kinetics and Isotherms

The adsorption kinetics experiments used the BMIGA material at the optimized dosage of 0.20 g L^–1^. The adsorbent was added to 250.00 mL Erlenmeyer flasks containing 20.00 mL of Pb­(II) solution with an initial concentration of 200.00 mg L^–1^, adjusted to pH 6.0. The suspensions were maintained under continuous agitation at 100 rpm. Sampling was performed at regular intervals from 10 min to 24 h. The adsorption isotherms were determined using analogous experimental conditions, in 250.00 mL Erlenmeyer flasks, at pH 6.0 and under agitation at 100 rpm, varying only the initial concentration of the Pb­(II) solution between 50.00 and 800.00 mg L^–1^. The experiments were performed at temperatures of 25.0, 30.0, 35.0, and 40.0 °C. The adsorption kinetics were analyzed using the pseudo-first-order (PFO), pseudo-second-order (PSO), Elovich, and intraparticle diffusion models. Equilibrium data were interpreted using the Langmuir, Freundlich, Temkin, and Dubinin–Radushkevich isotherm models. Detailed descriptions of the model fitting procedures and the corresponding parameters are provided in Sections S2 and S3 of the Supporting Material.

### 2.6 Adsorption Thermodynamics

Thermodynamic parameters including the Gibbs free energy change (Δ*G*°), enthalpy change (Δ*H*°), and standard entropy change (Δ*S*°) were estimated by fitting of the experimental adsorption data to appropriate models.^44^ The determination of these parameters enables evaluation of the spontaneity of the process and its endothermic or exothermic nature.^45^ The equilibrium constant *k* was defined as *k* = *q*_e_/*C*_e_. The Van’t Hoff equation is given by ln *k* = −Δ*H*°/(*RT*) + Δ*S*°/*R*. The values of Δ*H*° and Δ*S*° were calculated using the Van’t Hoff method, based on the slope and intercept of the linear plot obtained from the correlation between ln *k* and 1/*T*,^46^ as described by eq ([Disp-formula eq4]). The standard Gibbs free energy change (Δ*G*°) was calculated using eq ([Disp-formula eq5]), where (*R*) is the universal gas constant and (*T*) is the absolute temperature.^3^

4 $$\ln k=\frac{\Delta H^{{}^{\circ}}}{\mathrm{RT}}+\frac{\Delta S^{{}^{\circ}}}{R}=\frac{q_{e}}{C_{e}}$$

5 $$\Delta G^{{}^{\circ}}=-\mathrm{RT}\ln k$$

### 2.7 Adsorption Selectivity and Regeneration of the Adsorbent

The influence of coexisting cations and anions on the adsorption of Pb­(II) was evaluated using 5.00 mmol L^–1^ of NaCl, CaCl_2_, KNO_3_, and NaOAc, together with Pb­(II) at a concentration of 200.00 mg L^–1^, at pH 6.0 and 25 °C, and a BMIGA dosage of 0.25 g L^–1^, under agitation at 150 rpm for 3 h.

After Pb­(II) adsorption, the spent adsorbent was regenerated by immersion in 0.10 mol L^–1^ HNO_3_ under continuous agitation at 150 rpm for contact times of 1 and 3 h. Subsequently, the samples were washed repeatedly with deionized water until the supernatant reached approximately pH 7.0. The adsorption–desorption procedure was repeated for three consecutive cycles for each contact time.

### 2.8 Column Studies

A glass column with diameter of 30.0 cm was packed with 1.50 g of BMIGA, forming a bed height of 7.0 cm. Polystyrene beads were placed below the packed bed to prevent loss of the material (Figure S2). Pb­(II) ion solutions prepared at different concentrations (400.00, 600.00, and 800.00 mg L^–1^), at pH 6.0 and 25.0 °C, were pumped through the bed at flow rates of 1.00, 1.50, and 2.00 mL min^–1^, respectively. Aliquots were collected continuously, at a constant rate.

The efficiency of the fixed-bed column was evaluated based on breakthrough curves constructed from the *C*_i_/*C*_0_ vs *t* profile. The total amount of Pb­(II) ions adsorbed in the column (*q*_total_) and the column adsorption capacity (*q*_e_) were determined using eqs ([Disp-formula eq6]) and ([Disp-formula eq7]),^48^ where *Q* is the flow rate (mL min^–1^) and *m* is the mass of adsorbent (g).

6 $$q_{\mathrm{total}}=\frac{Q}{1000}\int_{t=0}^{t=t_{total}}C_{\mathrm{ad}}=\frac{Q}{1000}\int_{t=0}^{t=t_{total}}\left(C_{0}-C_{t}\right)d_{t}$$

7 $$q_{e}=\frac{q_{\mathrm{total}}}{m}$$

The results were fitted using the mathematical models of Thomas, Yoon-Nelson, and Clark, to obtain additional information about the fixed-bed adsorption process (Section S4).

## 3 Ecotoxicological Studies

*D. similis* was cultured at the Laboratory of the Aquatic Ecosystems Studies Group (GEEA) of the Federal University of Sergipe, following the guidelines established by ABNT NBR 12713:2022 for ecotoxicological assessments. The organisms were maintained in reconstituted water with total hardness ranging from 42.00 to 48.00 mg L^–1^ (as CaCO_3_), pH between 7.0 and 7.6, and temperature controlled at 23 ± 2 °C under a 12 h light/12 h dark photoperiod (12L:12D). The cultures were fed three times per week with the microalga *Raphidocelis subcapitata* (10^6^ cells mL^–1^), supplemented with Vitormonio (1.00 mL L^–1^). Culture sensitivity was evaluated monthly through acute toxicity tests using sodium chloride (NaCl) as the reference substance (Figure S3).

Acute toxicity assays with *D. similis* were conducted in accordance with ABNT NBR 12713:2022, exposing the organisms to 10.00 mL of solutions containing Pb­(II) ions at five concentrations ranging from 1 to 60 μg L^–1^. Five neonates (aged <24 h) were placed in each vessel, with four replicates per concentration, and exposed during a period of 48 h. The temperature was controlled at 23 ± 2 °C, a 12L:12D photoperiod was applied, and no feeding was performed during the experimental period. The results were expressed as EC_50.48h_. The 48 h EC_50_ value was determined using the Trimmed Spearman Karber method Hamilton et al.,^49^ and the corresponding 95% confidence interval was also calculated.

After the adsorption of Pb­(II) ions at an average concentration obtained from the EC_50.48h_, using the BMIGA adsorbent (0.25 g L^–1^) and pH values ranging from 6.0 to 7.5, the phases were separated by centrifugation at 4000 rpm for 10 min. The supernatants obtained after adsorption were then used in assays with *D. similis*. A control assay was performed in the absence of Pb­(II) ions. The solution pH values were recorded at the beginning and end of the assays. The Pb­(II) concentrations were quantified by atomic absorption spectrophotometry (AA-700, Shimadzu), since the levels were below the quantification limit of the UV–vis spectrophotometric method. All the assays were performed in triplicate.

## 4 Electrochemistry after Adsorption

The electrochemical analyses were performed using a potentiostat/galvanostat (PGSTAT M204, Metrohm Autolab), controlled with NOVA 2.1.8 software, and a 70.00 mL electrochemical cell. A conventional three-electrode system was employed, consisting of a reference electrode (Ag/AgCl in 3.00 mol L^–1^ KCl), a platinum wire (2.00 mm) as the counter electrode, and a carbon paste working electrode (CPE) obtained using a graphite: mineral oil mixture (70:30 ratio). In addition, a modified CPE was produced with the activated biomass (65:30:5). The current densities reported in this study were calculated by dividing the measured current by the geometric area of the working electrode. The potentials were calibrated to reversible hydrogen electrode (RHE) values using eq ([Disp-formula eq8]).

8 $$E\left(\mathrm{RHE}\right)=E\left(vs\frac{\mathrm{Ag}}{\mathrm{AgCl}}\right)+0.197+0.0592\times pH\;\left(V\right)$$

Linear sweep voltammetry (LSV) in the potential range 1.20-2.20 V vs RHE was used to determine the oxygen evolution overpotential, at a current density of 10 mA cm^–2^, with 1.00 mol L^–1^ KOH as the electrolyte. Energy for the electrochemical system was supplied by a photovoltaic energy source installed at the Laboratory of Corrosion and Nanotechnology (LCNT-NUPEG). This solar power system consisted of a Jinko JKM565N-72HL4-V 565 W solar panel, a GROWATT OFF-GRID SPF5000ES 5 kVA 220 V inverter, and a MOURA 12MS234 stationary solar battery (12 V, 220 Ah).

## 5 Results and Discussion

### 5.1 Characterization

The structural and chemical characterizations of the material were described previously in an earlier study by the same research group.^37^ The same characterization methodologies were employed here, but highlighting the aspects directly affecting the new process.

### 5.2 Adsorption Experiments

The effect of pH on the adsorption of Pb­(II) ions by BMIGA is shown in Figure 1a. The adsorption percentage was lower at pH 2.0, due to electrostatic repulsion caused by the presence of oxygenated functional groups, which became protonated as the concentration of H^+^ ions increased, evidencing competition between the H^+^ and Pb­(II) ions.^50^ As the pH increased, Pb­(II) removal became more efficient, reaching 46.79% at pH 8.0. However, precipitation of Pb­(II) as hydroxides may occur at higher pH,^51,52^ so pH 6.0 was selected in the adsorption studies. While Pb­(II) adsorption involves the retention of ions on the adsorbent surface via physicochemical interactions with active sites, precipitation refers to the formation of insoluble phases such as Pb­(OH)_2_(s) in solution and is not necessarily dependent on adsorption onto the solid surface.

**Figure 1:**
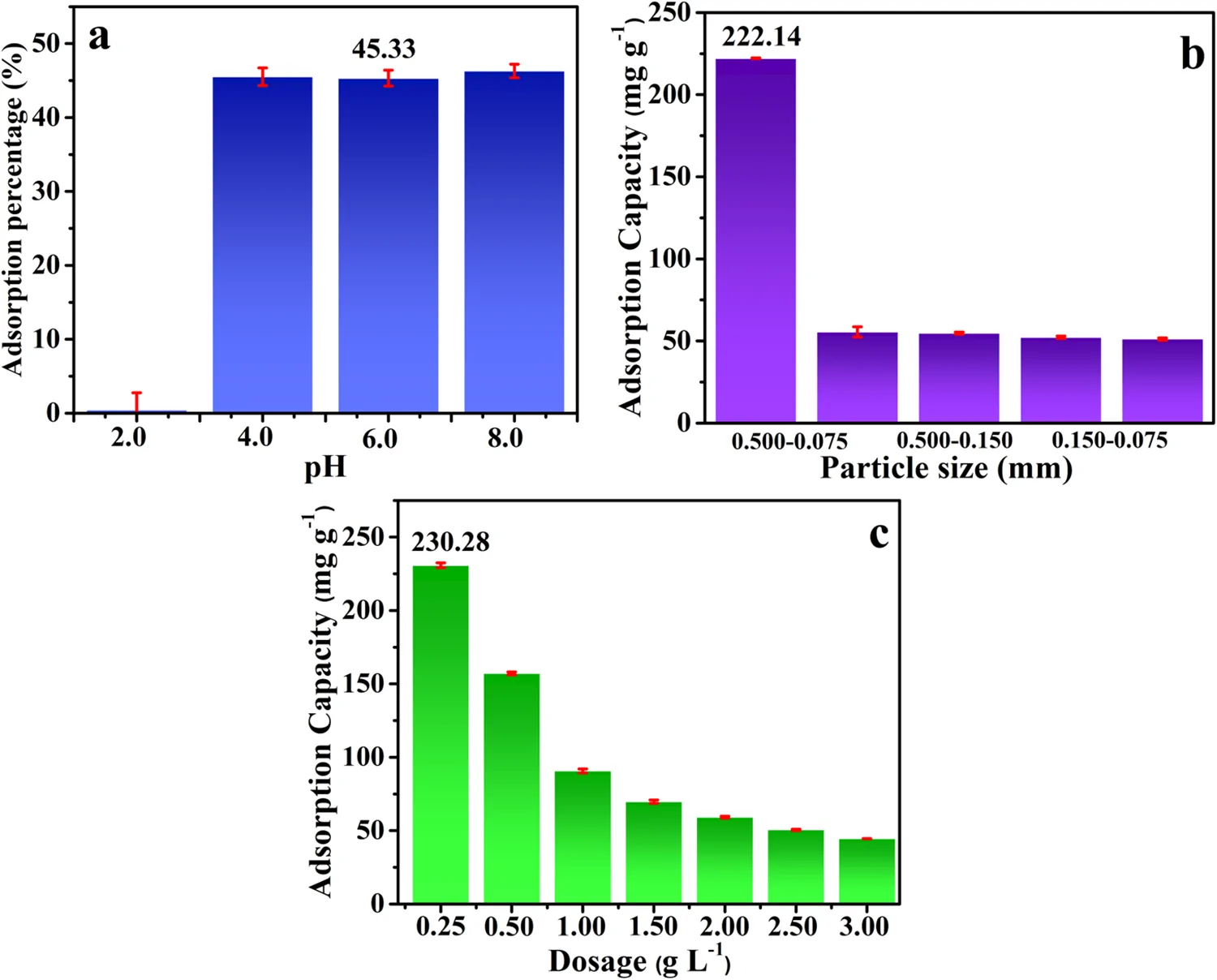
Effects of pH (a), adsorbent particle size (b), and adsorbent dosage (c) on the adsorption capacity of BMIGA for Pb­(II) ions. Experimental conditions: pH 6.0, Pb­(II) ions concentration of 200.00 mg L^–1^, and initial adsorbent dosage of 1.00 g L^–1^.

Speciation studies conducted at Pb­(II) concentrations close to those used in the present work (200 mg L^–1^) indicate that hydrolyzed lead species become increasingly significant with rising pH, with the onset of Pb­(OH)_2_ precipitation potentially occurring near pH 8.0.^53^ Madlangbayan et al.^54^ further reported that pH 8.0 favors the precipitation of lead hydroxide nitrate phases due to enhanced formation of hydrolyzed species and particle coagulation. Additionally, Quang et al.^55^ showed that Pb^2+^ predominates at pH ≤ 6, whereas hydrolyzed species (PbOH^+^, Pb­(OH)_2_, Pb­(OH)_3_^‑^) become increasingly relevant at higher pH values. Therefore, although the higher removal observed at pH 8.0 may be partly attributed to enhanced adsorption, the contribution of hydrolysis and precipitation phenomena cannot be disregarded. Evaluation of the non-activated precursor material showed inferior performance, compared to the activated material. The low removal efficiency could be explained by the high inorganic fraction, as described previously,^37^ which could lead to competition for the active sites, since many inorganic constituents were likely to be present in cationic forms. These findings highlight the importance of the activation step for optimizing performance in the removal of Pb­(II) ions.

The results for the effect of BMIGA particle size on the adsorption capacity for Pb­(II) ions (Figure 1b) showed that the highest adsorption capacity was achieved with particles in the size range from 0.500 to 0.075 mm. Although particle aggregation has been reported in the literature as a possible phenomenon affecting very fine adsorbent particles, no direct evidence of aggregation was obtained in the present study.^56^ Therefore, the lower adsorption performance observed cannot be conclusively attributed to this mechanism and may result from a combination of factors influencing mass transfer and accessibility of adsorption sites. Morphological and EDS analyses of the material after adsorption showed smoother surface features, associated with the presence of lead ions (Figure S4).^1^ Given these results, particles in the 0.500–0.075 mm size range were used in the dosage, kinetics, and isotherm assays.

Evaluation of the effect of adsorbent dosage (Figure 1c) showed that increase of the adsorbent mass from 0.25 to 3.00 g L^–1^ led to a decrease of the Pb­(II) adsorption capacity from 230.20 to 44.48 mg g^–1^. The corresponding Pb­(II) removal percentages for each adsorbent dosage have been included in Table S5 of the Supplementary Material. Although the adsorption capacity (*q*_e_) decreased with increasing adsorbent dosage, the Pb­(II) removal efficiency increased as the adsorbent dosage increased. This behavior is commonly observed in adsorption systems because a higher adsorbent mass provides a greater number of available adsorption sites, enhancing Pb­(II) uptake from solution. In contrast, the amount adsorbed per unit mass decreases due to the partial utilization of the available adsorption sites at higher dosages. Therefore, the selected dosage represents a compromise between adsorption capacity and overall removal efficiency. Evaluation of the effect of adsorbent dosage (Figure 1c) showed that increase of the adsorbent mass from 0.25 to 3.00 g L^–1^ led to a decrease of the Pb­(II) adsorption capacity from 230.20 to 44.48 mg g^–1^. This decrease was associated with rapid saturation of the activated sites, which increased competition among the adsorbent particles and reduced the adsorption efficiency.^57^

### 5.3 Adsorption Kinetics, Isotherms, and Mechanism

The mechanism of adsorption of Pb­(II) ions onto the chemically activated material was investigated using kinetic and isotherm studies. The adsorption kinetics of Pb­(II) ions reached equilibrium within 3 h (Figure 2a). Similar equilibrium times of approximately 3 h have also been reported for Pb­(II) adsorption onto other adsorbent materials.^58,59^ The adsorption isotherms (Figure 2b) indicated that the adsorption system became less favorable with increasing temperature. The maximum adsorption capacity was 222.14 mg g^–1^ at 25.0 °C. Other studies employing adsorbents for Pb­(II) removal also found that chemical modification with sulfuric acid enhanced the maximum adsorption capacity, attributed to surface functionalization and increased surface area.^58,60^ A comparison of different adsorbent materials for Pb­(II) uptake is provided in Table S4. The sulfuric acid-activated *I. edulis* biomass (222.14 mg g^–1^) exhibited performance comparable to or superior to that of adsorbents produced through sulfuric acid modification. The developed material showed a higher adsorption capacity than sulfuric acid-treated pumpkin peel biomass (178.6 mg g^–1^), sulfuric acid-activated bulrush biomass (32.6-44.6 mg g^–1^), and *Dypsis lutescens* leaf biomass associated with *Penicillium oxalicum* (97.85 mg g^–1^), being surpassed only by sulfuric acid-treated and pyrolyzed *Euphorbia rigida* biomass (279.72 mg g^–1^).^53,61,62^ Furthermore, BMIGA maintained removal efficiency at pH 4 and 6, which corresponds to the pH range employed in studies involving sulfuric acid-activated biomasses, demonstrating consistent and competitive performance under conditions comparable to those reported in the literature.^63,64^

**Figure 2:**
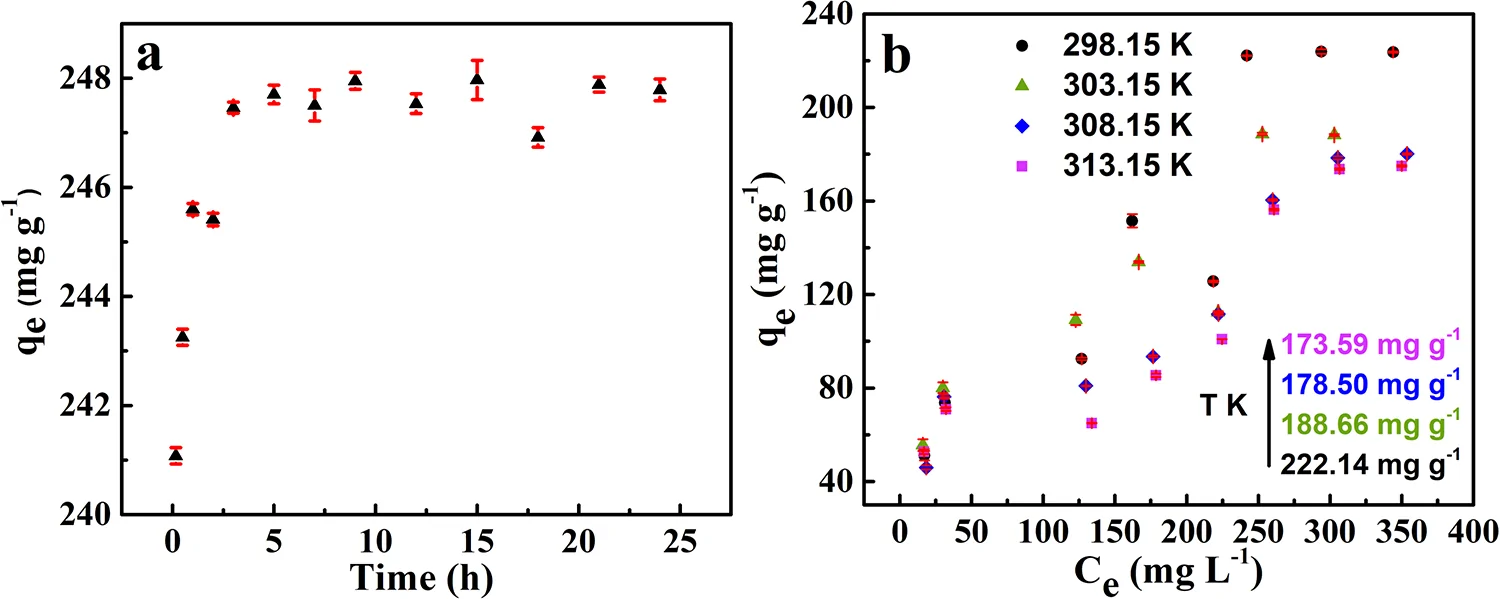
Adsorption kinetics (a) and isotherms (b) of Pb­(II) adsorption onto BMIGA. Experimental conditions: pH 6.0, initial Pb­(II) concentration of 200.00 mg L^–1^ for the kinetic experiments, and temperatures ranging from 298.15 to 313.15 K for the isotherm experiments.

The results showed that the chemically activated BMIGA had a higher adsorption capacity for Pb­(II), compared to adsorbents based on carbon nanotubes,^65^ iron-containing nanoparticles,^66^ and synthetic gels.^52^ Hence, BMIGA can be considered a promising alternative material for the treatment of Pb­(II)-contaminated effluents, suitable for large-scale adsorption processes, with the advantages of being derived from a renewable source and requiring only a simple chemical activation with sulfuric acid.

Although these adsorbents demonstrated satisfactory Pb­(II) removal efficiencies, the studies summarized in Table S4 were primarily limited to batch adsorption experiments. None of them investigated the performance of the adsorbents under continuous-flow conditions using fixed-bed columns, evaluated the ecotoxicological safety of the treated effluents, or explored the post-adsorption valorization of the Pb-loaded materials. In contrast, the present work extends beyond conventional adsorption studies by integrating fixed-bed column operation, ecotoxicological assessment using *Daphnia similis*, and the reuse of the spent adsorbent as an electrocatalyst for the oxygen evolution reaction (OER). This integrated approach enhances the practical applicability and sustainability of the proposed material, providing additional value within the framework of circular economy principles.

The adsorption mechanism of Pb­(II) ions onto BMIGA was investigated using kinetic and isotherm models. The adsorption kinetics were evaluated using the pseudo-first-order (PFO), pseudo-second-order (PSO), intraparticle diffusion (ID) and Elovich models.^67^ The equilibrium adsorption data were analyzed using the Langmuir, Freundlich, Dubinin–Radushkevich (DR) and Temkin isotherm models.^68^ The fitted curves for the kinetic and isotherm models are presented in Figures S5 and S6, respectively. The corresponding parameters obtained from these models are summarized in Tables S6 and S7.^69^ The mechanism of adsorption of Pb­(II) ions in the presence of the BMIGA adsorbent was investigated using adsorption kinetics and isotherm models.

The pseudo-second-order (PSO) kinetic model provided the best fit to the experimental data, exhibiting the highest correlation coefficient (*R* ^2^) among the evaluated models, along with the lowest chi-square (χ^2^) and root mean square error (RMSE) values. These results suggest that Pb­(II) adsorption kinetics is influenced by interactions between the adsorbate and the active sites of the adsorbent.^70^ However, it is important to emphasize that the PSO model is empirical in nature, and its good agreement with experimental data alone does not provide direct evidence of a chemisorption mechanism.^71^

The Freundlich model provided the best description of the equilibrium data, suggesting that Pb­(II) adsorption occurs on a heterogeneous surface with adsorption sites of different energies.^72^ The Freundlich exponent (nF) varied from 1.67 to 2.43, indicating favorable adsorption of Pb­(II) onto BMIGA.^73^ Furthermore, the corresponding 1/nF values (0.41-0.59) were lower than 1, this indicates favorable adsorption and a heterogeneous surface character of the adsorbent, as described in the literature.^74^

Further elucidation of the mechanism for adsorption of Pb­(II) was obtained by XPS analysis (Figure 3), which confirmed the presence of oxygenated functional groups, such as carboxyl and hydroxyl groups. The structural and functional characteristics of the sulfuric acid-activated material have been discussed in detail previously,^37^ where the presence of carboxyl and hydroxyl functionalities was confirmed. After adsorption, the O 1s spectrum (Figure 3b) showed a peak corresponding to PbO (530.68 eV). Deconvolution of the C 1s spectrum revealed the presence of a C=O peak (287.48 eV), related to the acetate ion from lead acetate. Additional changes, such as shifts in the peak energies of the C–OH and C–O–C groups from 285.05 and 286.88 eV to 284.68 and 286.78 eV, respectively, could be attributed to interactions with Pb­(II) ions, indicating the formation of stable complexes. Figure 3d shows the peaks corresponding to the two electronic orbitals associated with Pb­(II), detected at 139.28 and 143.98 eV.^75^ The coexistence of these peaks demonstrated that the adsorption process on the adsorbent surface involved interactions with functional groups, suggesting the formation of complexes, as well as lead hydroxides and oxides.^76^

**Figure 3:**
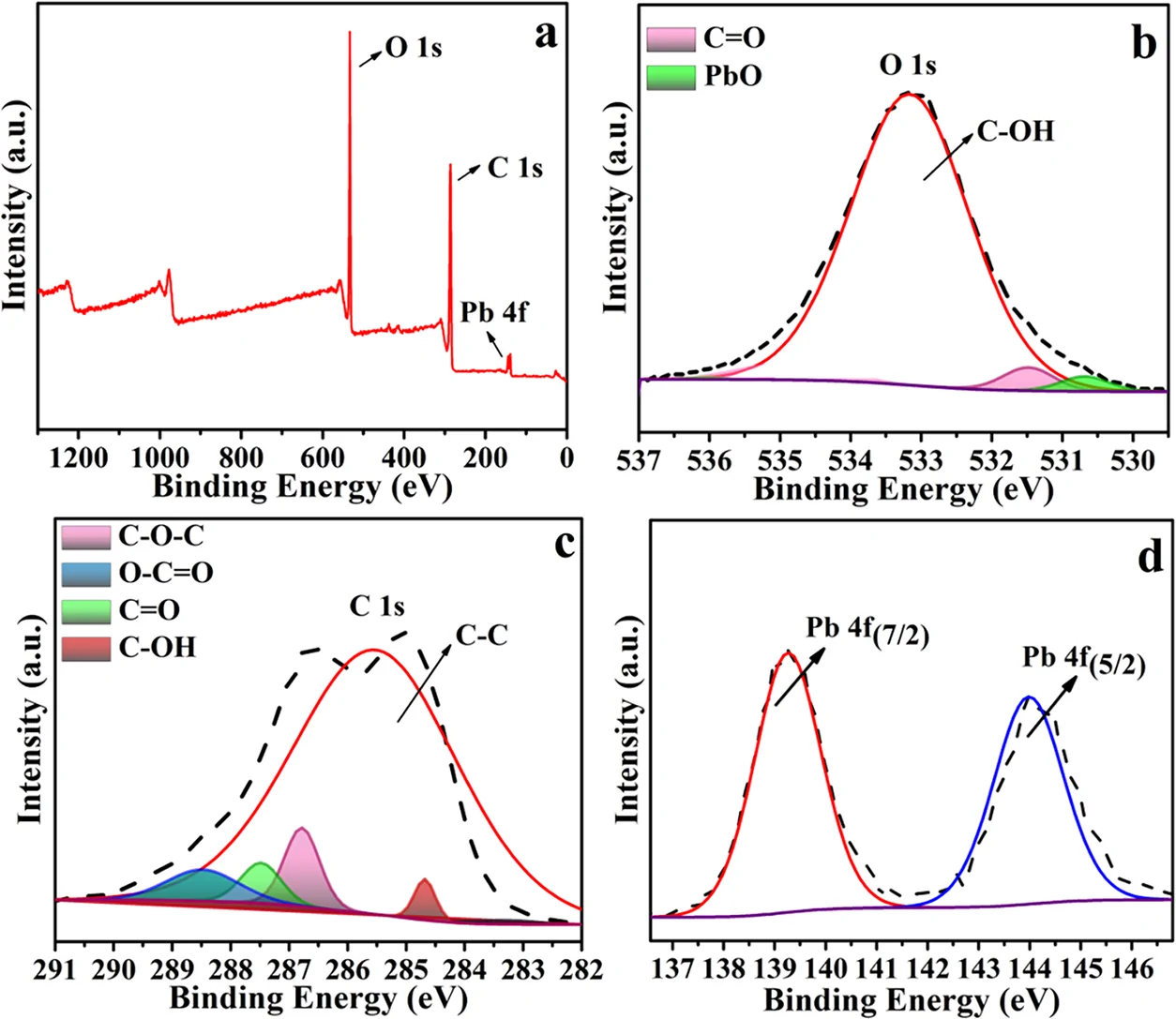
Total XPS spectrum of the activated biomass (BMIGA) (a) and deconvolutions of the C 1s, O 1s, and Pb 4f spectra (b–d, respectively).

### 5.4 Adsorption Thermodynamics

The results of the adsorption isotherm assays (Figure 2b) were used in the estimation of thermodynamic parameters. Figure 4 shows the relationship between the constant values (ln *k*) and temperature (1/*T*), which were used to obtain the enthalpy change (Δ*H*°) and entropy change (Δ*S*°) values, using eq ([Disp-formula eq4]). The Gibbs free energy change (Δ*G*°) was obtained using eq ([Disp-formula eq5]). Table 1 shows the thermodynamic parameters obtained for the adsorption of Pb­(II) ions on BMIGA.

**Figure 4:**
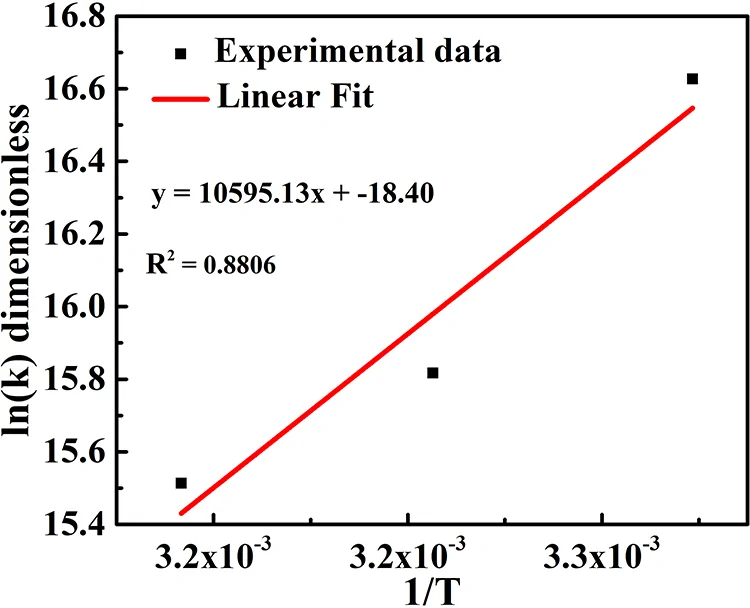
Fits obtained using adsorption isotherm models.

**Table 1:** Thermodynamic Parameters Obtained from the Isotherms for Adsorption of Pb­(II) Ions on BMIGA

adsorbent	temperature (K)	ln *k*	Δ*G*° (kJ/mol)	Δ*H*° (kJ/mol)	Δ*S*° (J/mol K)
BMIGA	298.15	15.74	–39.02	–88.08	–153.00
	303.15	16.62	–41.90		
	308.15	15.81	–40.52		
	313.15	15.51	–40.38		

The results (Table 1) showed that the adsorption of Pb­(II) ions on the BMIGA adsorbent was spontaneous across the entire temperature range, as indicated by the negative values of the Gibbs free energy change (Δ*G*°). The negative enthalpy change (Δ*H*°) suggested that the adsorption process was exothermic, consistent with the adsorption isotherm results (Figure 2b), where increase of the temperature led to a decrease in the adsorption capacity. The relatively high magnitude of this parameter suggests a significant contribution of strong surface interactions during adsorption. This interpretation is consistent with the XPS analysis, which revealed Pb 4f signals and Pb–O species after adsorption, as well as the involvement of oxygen-containing functional groups. These findings indicate that surface complexation and Pb–O bond formation contribute to Pb­(II) uptake, corroborating the thermodynamic evidence of strong adsorbate–adsorbent interactions. The negative entropy change (Δ*S*°) also indicated a reduction in system disorder during adsorption, probably due to interactions involving complexation and chemical bonding.^77^ Hence, chemical activation of the *I. edulis* biomass resulted in enhanced affinity of the BMIGA adsorbent for Pb­(II) ions, compared to the precursor material that showed no adsorption capacity.

### 5.5 Adsorption Selectivity and Regeneration of the Adsorbent

The selectivity of the adsorption process was evaluated in the presence of cations and anions commonly found in effluents. As shown in Figure 5a, the adsorption capacity for Pb­(II) decreased by approximately 49.00%, from 222.14 to 113.12 mg g^–1^, in the presence of Ca^2+^ and Cl^–^ ions, corresponding to a Pb­(II) removal efficiency of 50.99%. Although chloride ions can form aqueous complexes with Pb­(II), the chloride concentration employed in this study is relatively low and is not expected to significantly modify Pb­(II) speciation. This interpretation is supported by the NaCl experiment, in which only a slight decrease in adsorption capacity was observed despite the presence of chloride ions. Therefore, the pronounced reduction observed for CaCl_2_ is mainly attributed to competition between Ca^2+^ and Pb^2+^ for adsorption sites^78^ Other ions such as Na^+^, NO_3_^–^, and OAc^–^ (CH_3_COO^–^) had smaller effects on the adsorption capacity, yielding Pb­(II) adsorption capacities of 199.52, 162.19, and 176.18 mg g^–1^, which correspond to removal efficiencies of 89.75%, 72.88%, and 79.14%, respectively. These results indicate that the adsorbent maintained a high affinity for Pb­(II) even in the presence of competing ions. The lower interference observed for Na^+^, NO_3_^–^, and OAc^–^ can be attributed to their lower competitive potentials, resulting from their single charges and weaker interactions with the active groups on the adsorbent. In addition, the lower charge density of monovalent ions reduces their electrostatic affinity, contributing to less significant interference in the selective adsorption of Pb^2+^.^79^

**Figure 5:**
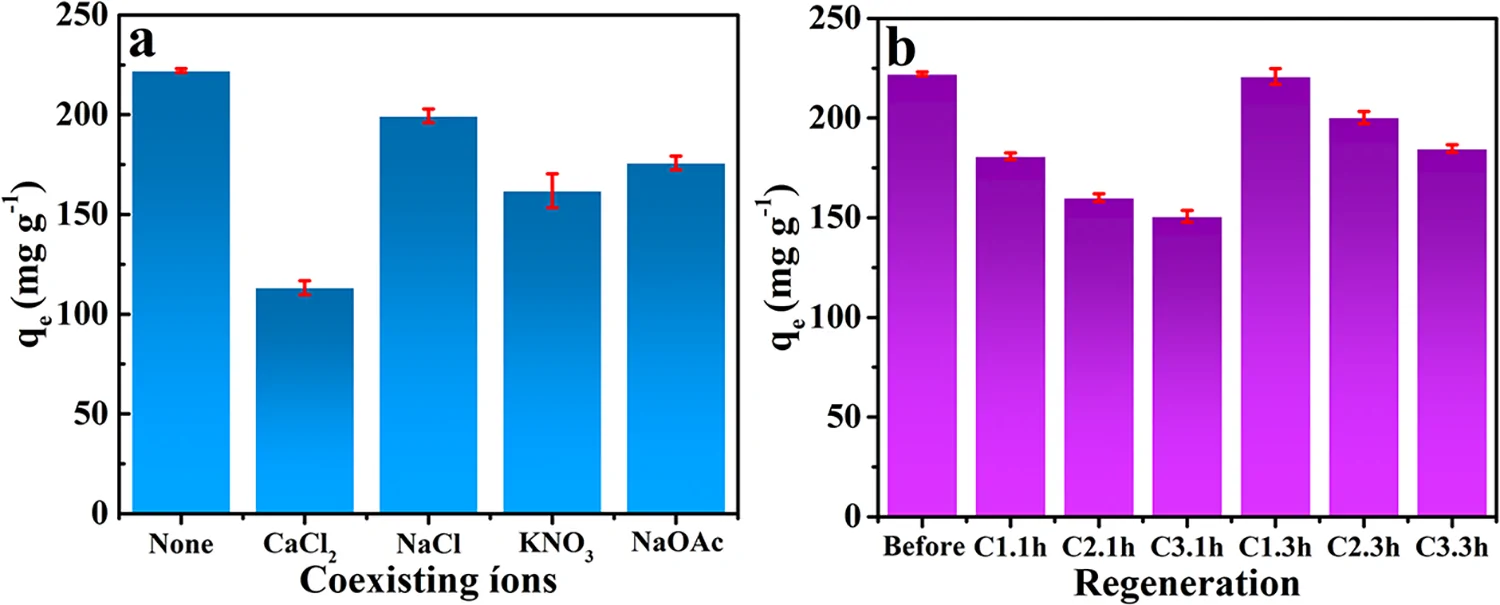
(a) Effect of coexisting ions on the adsorption process and (b) adsorption–desorption performance during regeneration cycles at contact times of 1 and 3 h.

As shown in Figure 5b, desorption efficiencies of 81.59%, 72.17%, and 67.38% were obtained in the regeneration tests conducted for 1 h. Higher performance was observed in the tests lasting 3 h, with 99.65% desorption in the first cycle, followed by decreases to 90.12% and 83.02% in the subsequent cycles. These results indicated that the adsorbent was able to maintain satisfactory regeneration capacity for up to three cycles, although with a progressive reduction in efficiency. This decline was due to partial loss of the active sites, related to degradation of the functional groups. The high desorption efficiency obtained using nitric acid reinforced its potential as an effective regenerating agent, enabling removal of the adsorbed Pb­(II) ions.^80^ Previous studies have suggested that lower desorption efficiencies observed in later cycles may be related to the saturation of active sites and the loss of oxygen-containing functional groups that are essential for adsorption interactions.^47,81^

### 5.6 Fixed-Bed Column Adsorption

The effects of initial concentration and flow rate on the adsorption process (Figure 6) were evaluated using Pb­(II) ion concentrations of 400.00, 600.00, and 800.00 mg L^–1^, with flow rates of 1.0, 1.5, and 2.0 mL min^–1^, respectively. The results showed that a higher initial concentration caused a reduction in the breakthrough time, which could be explained by rapid saturation of the active sites on the adsorbent.^82^ In addition, a higher flow velocity could result in higher residual concentrations in the solution and, consequently, lower removal efficiency.^83^ The number of bed volumes (BV) treated before breakthrough was calculated as the ratio between the treated solution volume at breakthrough and the bed volume (3.5 mL). The column treated approximately 159 BV at 400 mg L^–1^ and 146 BV at 600 mg L^–1^ before breakthrough, indicating a slight reduction in the service capacity of the bed at higher influent concentration. Overall, the results indicated the potential applicability of fixed-bed adsorption systems for the removal of Pb­(II) ions under conditions of lower initial concentrations and reduced flow rates, which allow the adsorbent material to be more efficiently utilized, suggesting the feasibility of scaling up the process for industrial applications.

**Figure 6:**
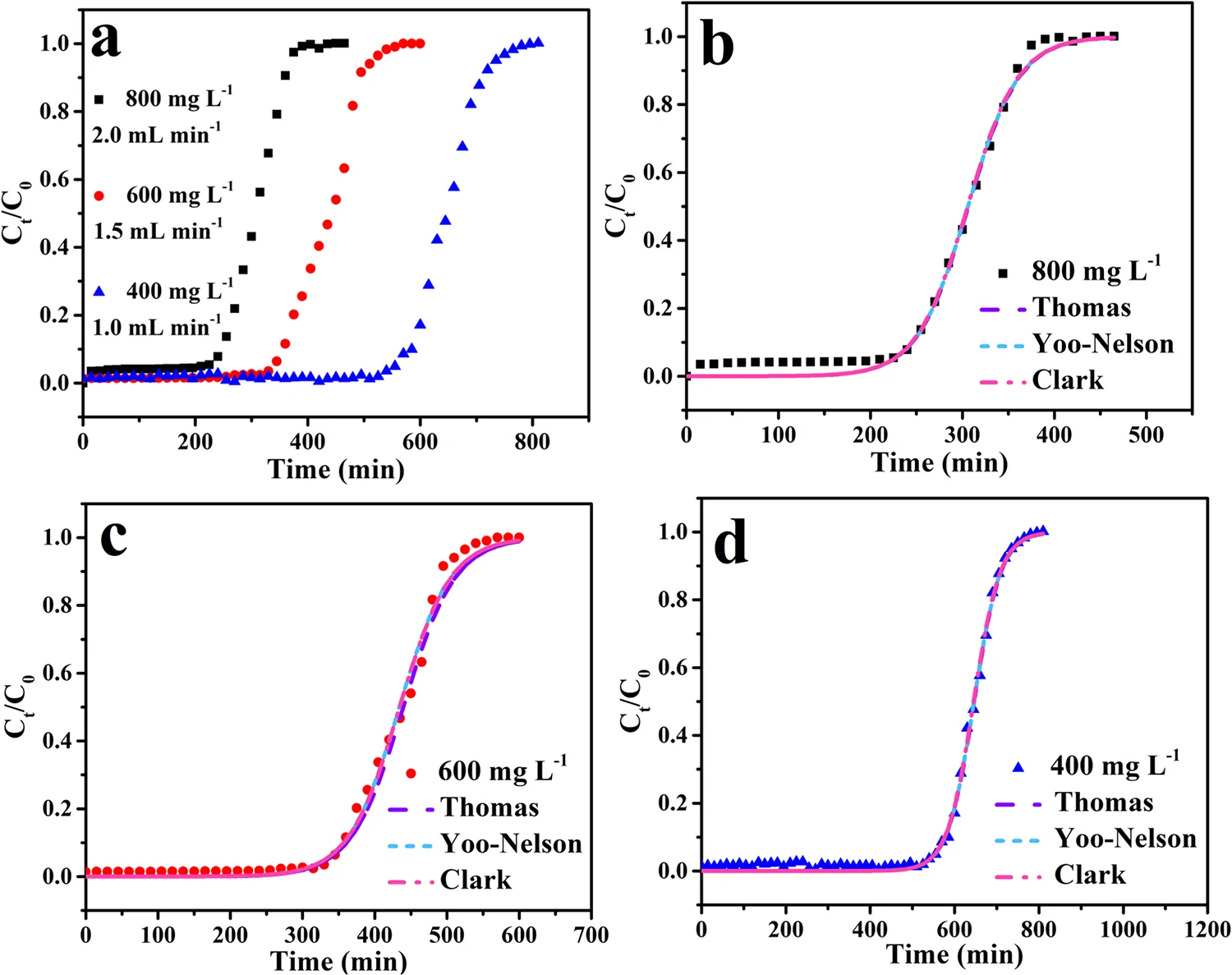
Column adsorption results: (a) effects of varying the initial Pb­(II) ion concentration and the flow rate; (b–d) fitting of adsorption models to the fixed-bed column data.

The statistical parameters shown in Table 2 (SSE, RMSE, and *R* ^2^) indicated that the Thomas and Yoon-Nelson models provided the best fits to the data for Pb­(II) adsorption in a fixed-bed column, especially at Pb­(II) concentrations of 400.00 and 600.00 mg L^–1^, with *R* ^2^ values close to 1 and low residual errors. Under these conditions, the two models showed nearly equivalent behavior, while the Clark model presented lower fitting quality.

**Table 2:** Error Parameters of the Thomas (TH), Yoon–Nelson (YN), and Clark (CL) Models Applied to Fixed-Bed Column Adsorption Data at Different Initial Pb­(II) Concentrations

metallic ion	concentration	errors	TH	YN	CL
Pb(II)		SSE	0.0160	0.0150	0.0165
	400.00 mg L^–1^	RMSE	0.0176	0.0174	0.0178
		*R* ^2^	0.9976	0.9977	0.9972
		SSE	0.0315	0.0314	0.0410
	600.00 mg L^–1^	RMSE	0.0288	0.0284	0.0371
		*R* ^2^	0.9948	0.9947	0.9845
		SSE	1.9150	1.8100	2.150
	800.00 mg L^–1^	RMSE	0.2570	0.2327	0.2629
		*R* ^2^	0.6835	0.6730	0.6550

At the highest concentration evaluated (800.00 mg L^–1^, with a flow rate of 2.0 mL min^–1^), significantly lower *R* ^2^ values were observed for all the models, indicating reduced representativeness of the fits. Under this condition, the lowest SSE and RMSE values were obtained with the Yoon-Nelson model, indicating its greater consistency. At 800.0 mg L^–1^, the substantial decrease in the fit quality (*R* ^2^ = 0.6730) evidences a loss of model reliability, likely associated with increased bed overloading and expansion of the mass transfer zone. Thus, the Yoon-Nelson model predictive performance within the practical operating range of 400–600 mg L^–1^, with *R* ^2^ values of 0.9977 and 0.9947, respectively.

Another parameter used to describe the adsorption process is the τ value, which is the time required for 50.00% of the solute to be adsorbed. The highest τ value obtained using the YN model was at the concentration of 400.00 mg L^–1^ (Table S8), associated with a longer breakthrough time.^84^ It was also observed that increasing the initial concentration (*C*_0_) significantly reduced the times for breakthrough (*t*_b_) and saturation (*t*_s_), indicating a shorter operational lifespan of the column. The mass transfer zone (MTZ) increased with *C*_0_, reflecting lower bed utilization efficiency. Conversely, the adsorption capacity (*q*_e_) was favored, increasing from 104.9 to 180.5 mg g^–1^, due to the higher concentration gradient. Similar findings have been reported by other researchers.^85–87^ Another parameter used to describe the adsorption process is the τ value, which is the time required for 50.00% of the solute to be adsorbed. The highest τ value obtained using the YN model was at the concentration of 400.00 mg L^–1^ (Table S7), associated with a longer breakthrough time.^84^ It was also observed that increasing the initial concentration (*C*_0_) significantly reduced the times for breakthrough (*t*_b_) and saturation (*t*_s_), indicating a shorter operational lifespan of the column. The mass transfer zone (MTZ) increased with *C*_0_, reflecting lower bed utilization efficiency. Conversely, the adsorption capacity (*q*_e_) was favored, increasing from 104.9 to 180.5 mg g^–1^, due to the higher concentration gradient.

The bed volume was 3.52 cm^3^, calculated from the column dimensions. Based on this volume, the empty bed contact time (EBCT) was 3.52 min for the 400 mg L^–1^ condition and 2.35 min for the 600 mg L^–1^ condition. In addition, the column treated approximately 158 bed volumes (BV) before breakthrough at 400 mg L^–1^ and 146 BV at 600 mg L^–1^, indicating a reduction in operational capacity with increasing influent concentration. The combination of a short EBCT with the treatment of more than 145 bed volumes before breakthrough demonstrates that the adsorbent can effectively remove Pb­(II) under continuous-flow conditions.^88^ The bulk bed density of BMIGA was 0.426 g cm^–3^, calculated from the adsorbent mass and the packed-bed volume, the relatively low bulk bed density obtained for BMIGA indicates a highly porous packed bed, which is advantageous for mass transfer and hydraulic performance because it favors fluid distribution and reduces flow resistance.^89^

### 5.7 Reuse of the Adsorbent in an Electrochemical Process

The water electrolysis process consists of two half-reactions: a cathodic reaction known as the hydrogen evolution reaction (HER), which involves the adsorption and desorption of hydrogen on an electroactive surface, and, simultaneously, at the anode of the electrochemical system, the oxygen evolution reaction (OER).^90^ Due to its complexity, the OER exhibits slow kinetics and generally requires high overpotentials. Therefore, research focused on the development of OER electrocatalysts has concentrated on increasing the catalytic activity of materials, to reduce the energy required to initiate the reaction. OER catalysts based on transition metals, such as those containing lead, present excellent catalytic activities and have therefore been extensively studied.^91^

In addition to their widespread use in energy storage devices such as Pb–C batteries and lead-acid batteries,^92^ lead-based structures have been used as electrocatalysts for electrochemical ozone production,^93^ CO_2_ reduction,^94^ and the oxygen evolution reaction itself.

In the present work, after the adsorption process, the material containing lead ions was used as an electrocatalyst for the OER. Analysis using linear sweep voltammetry (LSV) (Figure 7) showed that the electrode modified with the BMIGA-Pb sample presented superior electrocatalytic activity, compared to the unmodified electrode. This improvement was confirmed by the lower potential required to reach a current density of 10 mA cm^–2^ (1.59 V vs RHE), relative to the unmodified electrode (1.90 V vs RHE).

**Figure 7:**
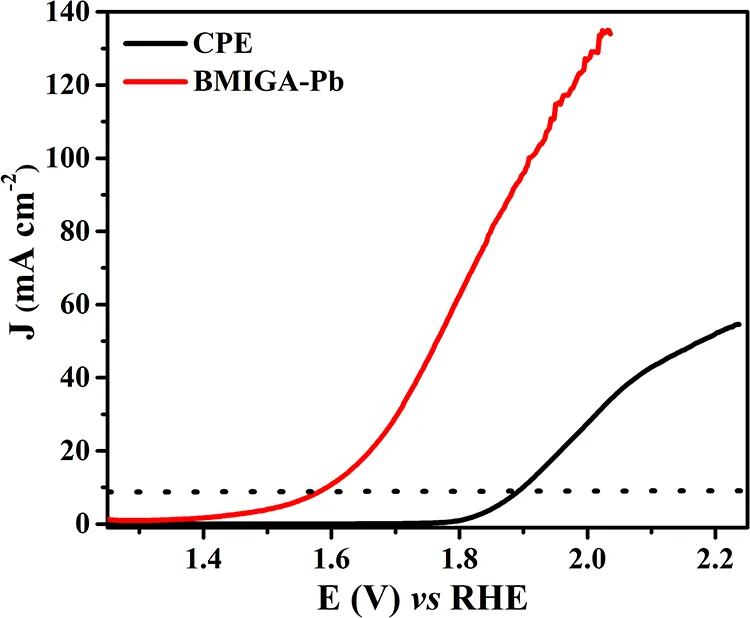
Linear sweep voltammograms obtained for the oxygen evolution reaction activity of the carbon paste electrode (CPE) and the modified carbon paste electrode (BMIGA-Pb) in 1.00 mol L^–1^ KOH solution (ν = 5.0 mV s^–1^).

The improvement shown in Figure 7 could be attributed to the presence of functional groups in the carbon-based material, as confirmed by FTIR and XPS analyses. In addition, the incorporation of lead into the carbon structure facilitated electron transfer during the OER, which lowered the activation energy of the reaction and, consequently, the overpotential required.^95,96^ The results showed that after use for adsorption, the material could be employed for the oxygen evolution reaction (OER), as an alternative end-use strategy, avoiding the formation of potentially toxic byproducts associated with disposal methods such as landfill or incineration.^97,98^ Therefore, although this approach involves the reuse of a Pb-containing material, the immobilization of Pb species within a carbonaceous biomass-derived matrix provides a controlled pathway for pollutant recovery and material valorization. Further studies involving quantitative Pb leaching analysis after OER operation, using techniques such as ICP-OES/ICP-MS or electrochemical sensing approaches, are important to confirm the long-term environmental applicability and safety of this strategy.

Furthermore, oxygen production is highly relevant, since it is essential in various industrial, environmental, and energy-related processes, including wastewater treatment, oxygenation of aquatic systems, and energy conversion in electrochemical devices.^99,100^ Therefore, repurposing the adsorbent material for the OER adds both functional and environmental value to the process, promoting a more sustainable and integrated approach.

### 5.8 Toxicity Evaluation

The exposure of *D. similis* to the Pb­(II) ion solutions resulted in an average EC_50.48h_ value of 13.60 μg L^–1^ (Table 3).

**Table 3:** Description and Results of Acute Toxicity Tests Using *D. similis* Exposed to Pb­(II)

parameters	data
Duration	48 h
pH	6.5–7.5
Contact concentrations	1.00–60.00 μg L^–1^
EC_50.48h_	13.60 ± 3.70 μg L^–1^
Coefficient of variation	27.24%
Stock solution	20.00 mg L^–1^

After the adsorption process, at an initial Pb­(II) concentration of 13.60 μg L^–1^, no acute toxic effects were observed in the organisms, confirming the effectiveness of BMIGA for Pb­(II) removal. In Brazil, CONAMA Resolution No. 430/2011 establishes a maximum permissible concentration of 0.50 mg L^–1^ for total lead in effluents. Furthermore, the Agency for Toxic Substances and Disease Registry (ATSDR) classifies lead as one of the most toxic and hazardous metals.^101,102^

The results of the EC_50.48h_ assays indicated a significant acute effect of the lead ions at low concentrations, revealing toxicity at levels approximately 37 times lower than the limit established by legislation. In some studies of effluent contamination in Brazil, Pb­(II) ion concentrations reached values between 0.03 and 0.09 mg L^–1^.^103–105^ In the study by Demarco et al., an aquatic macrophyte was used for the removal of heavy metals, with Pb­(II) at 0.18 mg L^–1^ observed in the Santa Bárbara stream (Brazil). Hence, *D. similis* can be used for the monitoring of effluents containing trace levels of Pb­(II) ions. After the adsorption process using BMIGA, *D. similis* did not exhibit acute effects, demonstrating the efficiency of the adsorption. The concentration of Pb­(II) in the treated samples was below the instrumental detection limit of the AAS system used (CRDL = 3 μg L^–1^, Shimadzu AA-7000). The residual Pb­(II) concentration was below 3 μg L^–1^, which is considerably lower than the EC_50.48h_ value obtained for *D. similis* (13.60 μg L^–1^). This supports the conclusion that Pb­(II) was effectively removed to concentrations that did not induce acute toxicity in the post-adsorption supernatants.

## 6 Conclusions

The results of this study demonstrated that sulfuric acid-modified *I. edulis* biomass (BMIGA) is an effective adsorbent for Pb­(II) removal from aqueous media, showing enhanced adsorption performance associated with increased surface area and oxygen-containing functional groups. Adsorption behavior was successfully described by kinetic and isotherm models, while the fixed-bed experiments indicated the feasibility of continuous operation for Pb­(II) removal. Among the evaluated models, the Yoon–Nelson model provided the best fit to the column adsorption data, indicating predictable dynamic behavior of the system. Ecotoxicological assays using *D. similis* confirmed the absence of acute toxicity in the treated effluent, supporting the effectiveness of the treatment process. Furthermore, the Pb-loaded material showed potential for reuse as an electrocatalytic material toward the oxygen evolution reaction, demonstrating an alternative strategy for the valorization of spent adsorbent. Overall, the findings indicate that BMIGA can be considered a promising low-cost material for Pb­(II) remediation, while its post-treatment reuse contributes to waste minimization and resource valorization.

## Supplementary Material



## Notes

UV–Vis spectra and calibration curve for Pb­(II); kinetic and adsorption isotherm model equations; fixed-bed column adsorption system and model equations; *D. similis* control test; morphology and EDS characterization before and after Pb­(II) adsorption; comparative Pb­(II) adsorption capacities; kinetic and isotherm model fits; kinetic, equilibrium, and fixed-bed column model parameters (PDF)
