# Supplementary material for: Sustainable Valorization of Pb(II)-Loaded Inga edulis Biomass: From Adsorption to Oxygen Evolution Electrocatalysis
Source: ACS Omega. 2026 Sep 9;11(37):55806–19. doi: 10.1021/acsomega.6c05335 (PMC13613927; doi:10.1021/acsomega.6c05335)
Supplement: Supplementary file 1 [file ao6c05335_si_001.pdf]

SUPPLEMENTARY MATERIAL

Sustainable Valorization of Pb(II)-Loaded *Inga edulis* Biomass: From Adsorption to Oxygen Evolution Electrocatalysis

Lucas dos S. Lima[a], Erica P. Fernandes[a], Andrea Novelli[b], Angel Tomé de L. Silva[b], Luiz Pereira da Costa[c], Marcos V. Quirino dos Santos[a], Jefferson A. Freitas[d], Eliana M. Sussuchi[a]*

[a]Research Group in Electrochemical Sensors and nano(Materials) - SEnM, Laboratory of Corrosion and Nanotechnology - LCNT, Graduate Program in Chemistry, Department of Chemistry, Federal University of Sergipe, Av. Marcelo Deda Chagas, 304, Rosa Elze, São Cristóvão, Sergipe, 49107-230, Brazil

[b]Aquatic Ecosystems Study Group - GEEA, Department of Environmental Engineering, Federal University of Sergipe, Av. Marcelo Deda Chagas, 304, Rosa Elze, São Cristóvão, Sergipe, 49107-230, Brazil

[c]Laboratory of Nanotechnology and Functional Materials - LANanMF, Department of Chemistry, Federal University of Sergipe, Av. Marcelo Deda Chagas, 304, Rosa Elze, São Cristóvão, Sergipe, 49107-230, Brazil

[d]Environmental Technology Laboratory - LATAM, Department of Environmental Engineering, Federal University of Sergipe, Av. Marcelo Deda Chagas, 304, Rosa Elze, São Cristóvão, Sergipe, 49107-230, Brazil

*Corresponding author address: Laboratory of Corrosion and Nanotechnology - LCNT, Graduate Program in Chemistry, Department of Chemistry, Federal University of Sergipe, Av. Marcelo Deda Chagas, 304, Rosa Elze, São Cristóvão, Sergipe, 49107-230, Brazil

*E-mail address: esmidori@gmail.com (Eliana Midori Sussuchi)

ORCID List

Lucas dos S. Lima: 0000-0002-6628-7599

Erica P. Fernandes: 0009-0000-8163-6278

Andrea Novelli: 0000-0002-5072-9647

Angel Tomé de L. Silva: 0009-0003-7289-8024

Luiz Pereira da Costa: 0000-0002-2632-6485

Marcos V. Quirino dos Santos: 0000-0002-0111-1699

Jefferson A. Freitas: 0000-0002-8120-734X

Eliana M. Sussuchi: 0000-0001-9425-0921

Section S1.

Using a flame atomic absorption spectrophotometer (double optical beam, spectral range 185–900 nm, Shimadzu, model AA-700). The calibration curve was prepared from a multielement standard solution (1000 ppm). For the quantification of Pb(II) ions by ultraviolet–visible spectroscopy (UV–Vis), the calibration curve was obtained at pH 6.0, using the maximum absorption wavelength at 208 nm (Fig. S1).

Fig. S1. (a) UV–Vis absorption spectra of Pb(II) ions at different concentrations (10–60 mg L^{-1}) at pH 6.0, showing the maximum absorption at 208 nm. (b) Corresponding calibration curve with linear fit (R^2 = 0.9992).

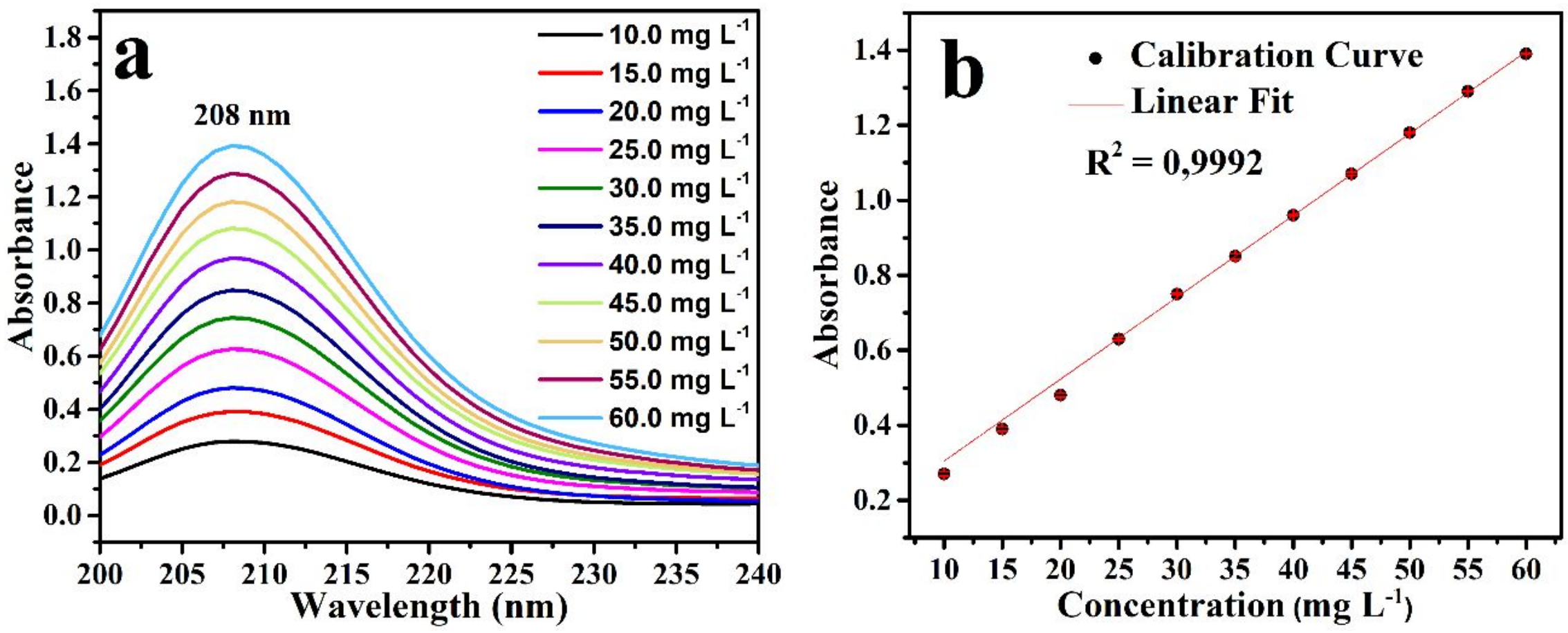

Section S2.

Kinetic modeling is essential for understanding how adsorbates interact with the active sites of an adsorbent over time. Different models have been proposed in the literature to describe adsorption rates and to elucidate the mechanisms involved. One of the earliest approaches is the **Pseudo-first-order (PFO) model**, initially formulated by Lagergren.[1] This model assumes that the adsorption rate is proportional to the number of unoccupied active sites on the adsorbent. Its mathematical expression is given in Equation

(1). Where k_1 (h^{-1}) denotes the rate constant, q_e (mg g^{-1}) is the adsorption capacity at equilibrium, q_t (mg g^{-1}) the adsorption at a given time t (h).

To overcome the limitations of the PFO model at longer contact times, Ho proposed the **Pseudo-second-order (PSO) model.**[2] This model considers that adsorption depends on the square of the number of available sites, and that the rate is directly related to the adsorbent capacity. Equation (2) presents its general form. The PSO parameters include k_2 (g mg^{-1} h^{-1}), q_e, q_t, and time t.

Another frequently applied model is the **Elovich equation**, originally developed for gas adsorption on solids.[3] It assumes that the adsorption rate decreases exponentially as coverage increases, reflecting surface heterogeneity. Its mathematical form is shown in Equation (3). Here, α (mg g^{-1} h^{-1}) represents the initial adsorption rate, and β (mg g^{-1}) is related to the extent of surface desorption.

The **Intraparticle Diffusion (ID) model**, introduced by Weber and Morris, describes adsorption as being controlled by diffusion of the solute within the pores of the adsorbent.[4] According to this model, adsorption capacity increases proportionally with the square root of time, as shown in Equation (4). In this case, qₜ is the adsorption capacity at time t, k_{ID} (mg g^{-1} $h^{-0.5}$) the intraparticle diffusion constant, and C_{ID} (mg g^{-1}) a parameter associated with the boundary layer thickness. The equations of the kinetic models are presented in Table S1.

Table. S1. Kinetic model equations.

Models	Equations
$\frac{d_{qt}}{dt} = k_1(q_e - q_t)$	(1)
$\frac{\mathbf{dq_t}}{\mathbf{d_t}} = \mathbf{k_2}\ (\boldsymbol{q_e} - \boldsymbol{q_t})^{\mathbf{2}}$	(2)
$\frac{d_{qt}}{d_t} = \alpha\ \exp(-\beta_{qt})$	(3)
$q_t = k_{ID}\sqrt{t} + C_{ID}$	(4)

Section S3.

Isotherm models are fundamental to describing how solutes interact with an adsorbent surface at equilibrium.[5] They provide insights into adsorption capacity, the nature of the adsorbent–adsorbate interactions, and whether the process occurs as monolayer or multilayer coverage.

One of the most widely applied models is the **Freundlich isotherm**, which assumes a heterogeneous surface with adsorption sites of different energies.[6] Unlike models that restrict adsorption to a monolayer, Freundlich's approach is suitable for multilayer adsorption. Its nonlinear expression is given in Equation (1). In this equation, K_F ($L^{1/nF}$ $mg^{1-1/n}$ g^{-1}) represents the Freundlich constant related to adsorption capacity, while *1/nF* indicates surface heterogeneity. q_e (mg g^{-1}) corresponds to the equilibrium adsorption capacity, and C_e (mg L^{-1}) is the solute concentration at equilibrium.

In contrast, the **Langmuir isotherm** is based on the assumption of a homogeneous surface with identical adsorption sites, all possessing equal binding energies.[7] Adsorption proceeds until a monolayer is formed, after which no further uptake occurs. The model is represented in Equation (2). Here, q_m (mg g^{-1}) is the maximum adsorption capacity, while K_L (L mg^{-1}) is the Langmuir constant related to the affinity between adsorbent and adsorbate.

The **Sips isotherm** combines characteristics of both Freundlich and Langmuir models, making it applicable to a broad range of adsorption systems, whether homogeneous or heterogeneous.[8] Its expression is given in Equation (3). Another widely employed model is the **Temkin isotherm**, which incorporates adsorbent–adsorbate interactions by assuming that the adsorption energy decreases linearly with coverage.[9] Equation (4) presents its general form. The Temkin parameters are q_e (mg g^{-1}), the equilibrium adsorption capacity; R (J mol^{-1} K^{-1}), the universal gas constant; T (K), the

absolute temperature; b, a constant related to the heat of adsorption; K_T (L mg^{-1}), the Temkin constant; and C_e (mg L^{-1}), the solute concentration at equilibrium.

Finally, the **Dubinin–Radushkevich (D–R) isotherm** is often used to evaluate adsorption processes involving microporous solids.[10] This model does not assume a homogeneous surface or constant adsorption potential but instead accounts for the energy distribution across the surface. The general form is presented in Equation (5). In this model, q_e (mol g^{-1}) is the adsorption capacity at equilibrium, q□ (mol g^{-1}) the theoretical maximum adsorption, and k the constant related to adsorption energy (mol^2 kJ^{-2}). The potential energy, ε, is calculated using Equation (6): where R = 8.314 J mol^{-1} K^{-1}, T is the temperature (K), and C_e is the equilibrium concentration of the adsorbate (mol L^{-1}). The equations of the adsorption isotherm models are presented in Table S2.

Table S2. Equações dos modelos de isoterma de adsorção.

Models	Equations
$q_e = k_F C_e^{1/nF}$	(1)
$q_e = \frac{q_m k_L C_e}{1 + k_L C_e}$	(2)
$q_e = \frac{q_{ms} k_s C_e^{ns}}{1 + k_s C_e^{ns}}$	(3)
$q_e = \frac{RT}{b} \ln(k_T C_e)$	(4)
$q = q_m \exp(-k_\varepsilon^2)$	(5)
$\varepsilon = RT \ln\left(1 + \frac{1}{C_e}\right)$	(6)

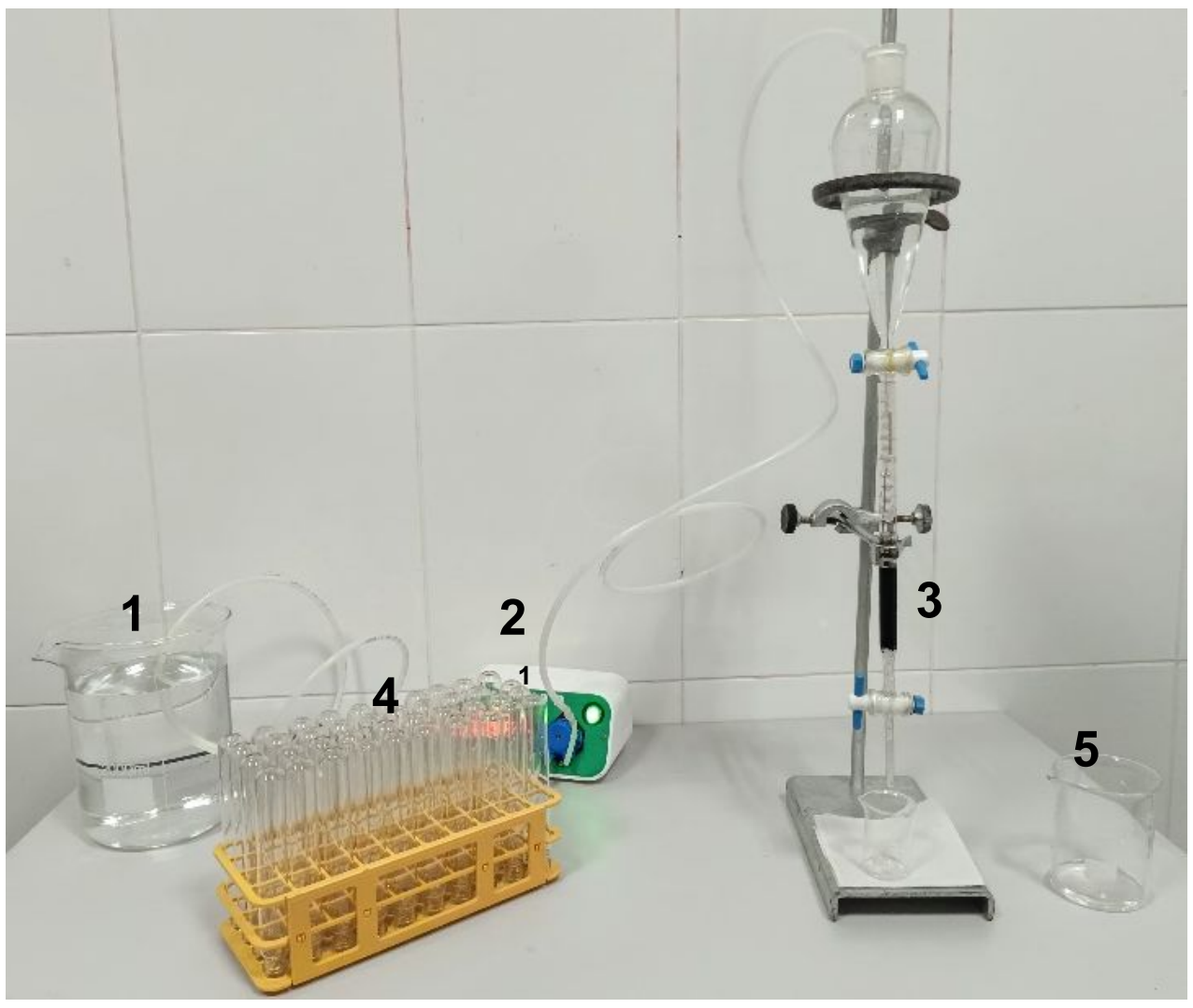

Fig. S2. Column adsorption system for the removal of Pb(II) ions: (1 – Pb(II) ion stock solution, 2 – peristaltic pump, 3 – glass column with a fixed-bed adsorbent, 4 – test tubes for aliquot collection, 5 – treated effluent reservoir).

Section S4.

Empirical models are used to accurately investigate the breakthrough curve. Among these models are the Thomas model, the Yoon–Nelson model, and the Clark model.[11,12]

The **Thomas model** is applicable to both favorable and unfavorable adsorption conditions. Traditionally, this model is used to determine the maximum adsorption capacity of an adsorbent in continuous systems.[11] The parameters in Equation 1 represent: C, metal concentration at a given volume (mg L^{-1} or mol L^{-1}); C_0, initial solution concentration (mg L^{-1} or mol L^{-1}); K_{TH}, Thomas constant; q_0, maximum solute concentration in the solid phase, i.e., maximum adsorption capacity (mg g^{-1}); m_s, mass of adsorbent (g); F_m, volumetric flow rate (L min^{-1}); and V_e, effluent volume from the column (mL).

The **Yoon–Nelson model** is based on a theoretical assumption that does not focus on the properties of the adsorbed material, on the adsorbent type, or on any physical characteristics of the adsorption bed.[13,14] The model assumes that the decreasing rate of adsorption is directly proportional to the adsorption of the adsorbed material and to the progression of the adsorbent [14]. In the Equation 2 of this model, K_{YN} represents the curvature degree of the breakthrough curve, and τ is the time required for 50% breakthrough.

The **Clark model** combines the Freundlich isotherm with mass transfer theory.[13] To apply the Clark model, the Freundlich isotherm parameter (n) must be determined; in this study, a value of 1.6701 was used. In Equation 3, A is the Clark model constant, r (mg $L^{-1} \times$ h) is the adsorption rate, and n is the Freundlich adsorption constant. Table 3 presents the equations of the models.

Table 3. Equations of the fixed-bed column models.

Models	Equations
$\frac{C_t}{C_0} = \frac{1}{1+e^{(k_{TH} q_0 m/Q - k_{TH} C_0 t)}}$	(1)
$\frac{C_t}{C_0} = \frac{1}{1+e^{k_{YN(\tau - t)}}}$	(2)
$\frac{C_t}{C_0} = \frac{1}{[1+ Ae^{-rt}]^{(\frac{1}{n-1})}}$	(3)

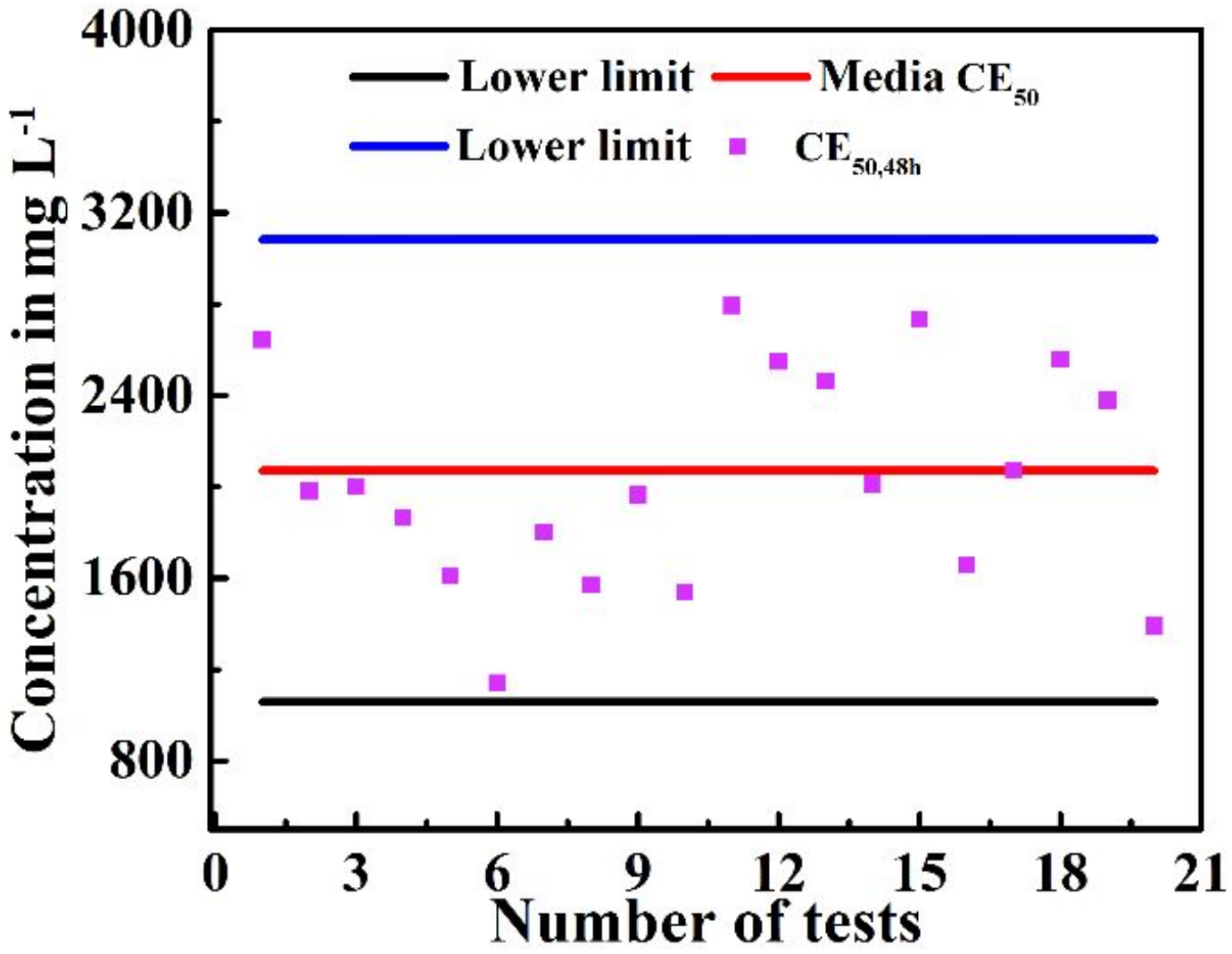

Fig. S3. Control table of the test organism *Daphnia similis* obtained through acute tests with the reference solution sodium chloride (NaCl): standard deviation of 505.9, coefficient of variation of 24.42 and lower and upper limits of 1059.80 and 3083.33 respectively.

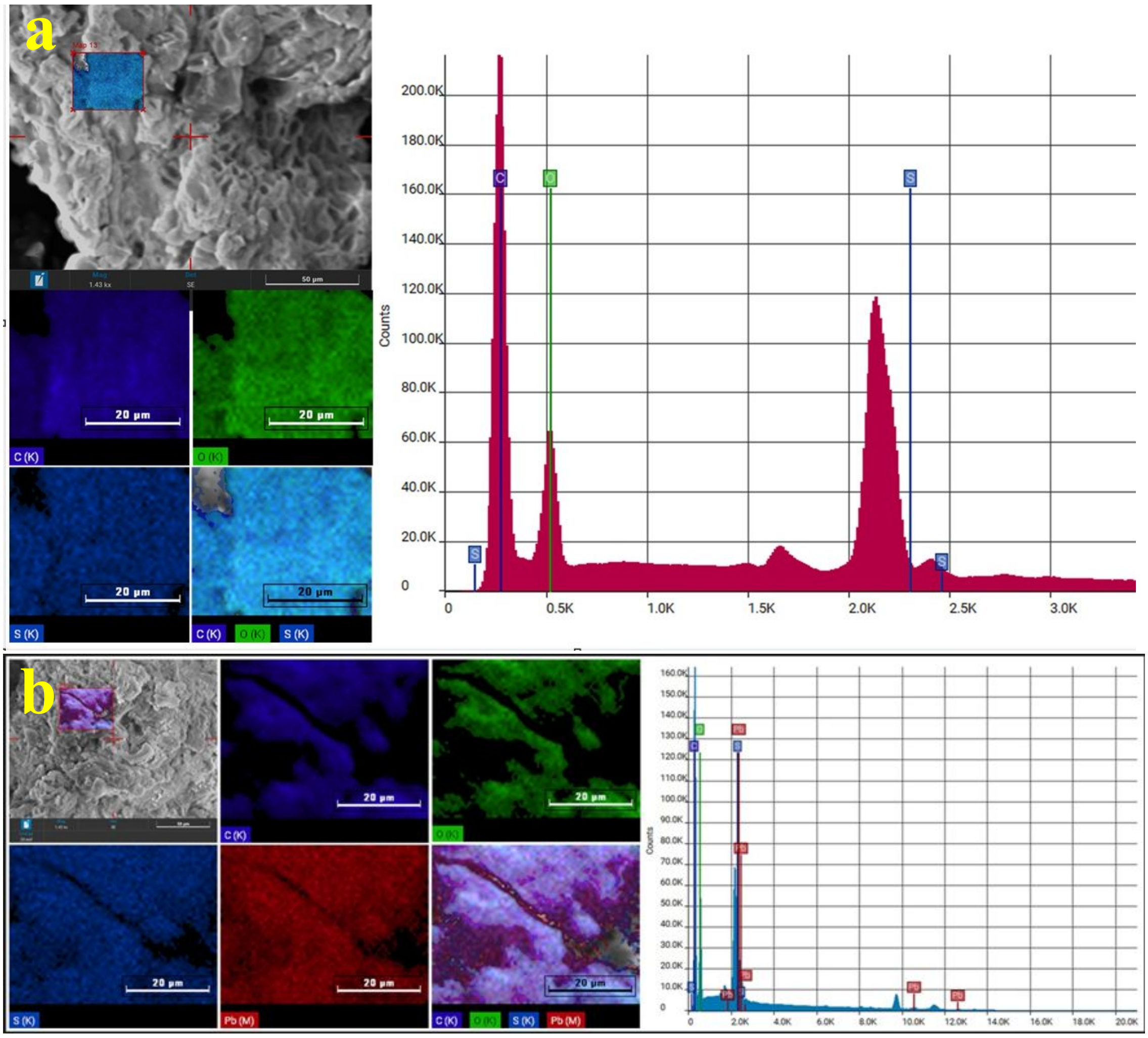

Fig. S4. Morphology and EDS before adsorption (a) and after adsorption of Pb(II) ions (b).

Table S4. Comparative studies of Pb(II) adsorption capacity with other adsorbents.

Adsorbent	q_e (mg g^{-1})	Adsorption condition	References
Sulfuric acid-treated and pyrolyzed Euphorbia rigida biomass	279.72	pH = 4.0<br>Dosage = 0.80 g L^{-1}<br>T = 313.15 K	[15]
Hazelnut and almond shells	28.10 and 8.08	pH = 6.0-7.0<br>Dosage = 0.40 g L^{-1}<br>T = 298.15 K	[16]
Sulfuric acid-activated biomass of Dypsis lutescens	97.85	pH = 5<br>Dosage = 3.2 g L^{-1}<br>T = 303.15 K	[17]
Pyrolyzed mixed biomass of potato and banana peels with TiO_2	7.80, 13.40 and 47.70	pH = 5.0<br>Dosage = 1.0 g L^{-1}<br>T = 308.15 K	[18]
Pyrolyzed biomass (900 °C)	91.80	pH = 5.0<br>Dosage = 0.16 g L^{-1}<br>T = 298.15 K	[19]
Sulfuric acid-activated bulrush biomass	32.6 and 44.6	pH = 5.0<br>Dosage = 0.10 g L^{-1}<br>T = 298.15 K	[20]
Olive leaf biomass	68.90	pH = 7.5<br>Dosage = 2.0 g L^{-1}<br>T = 293.15 K	[21]
Sulfuric acid-treated pumpkin peel biomass	178.6	pH = 5.0<br>Dosage = 0.15 g L^{-1}<br>T = 298.15 K	[22]
Dried inactivated fungal biomass (*Panaeolus papilionaceus*)	31.20	pH = 4.0<br>Dosage = 1.0 g L^{-1}<br>T = 298.15 K	[23]
**Biomass of *Inga edulis* activated with sulfuric acid**	**222.14**	**pH = 6.0**<br>**Dosage = 0.25 g L^{-1}**<br>**T = 298.15 K**	**This work**

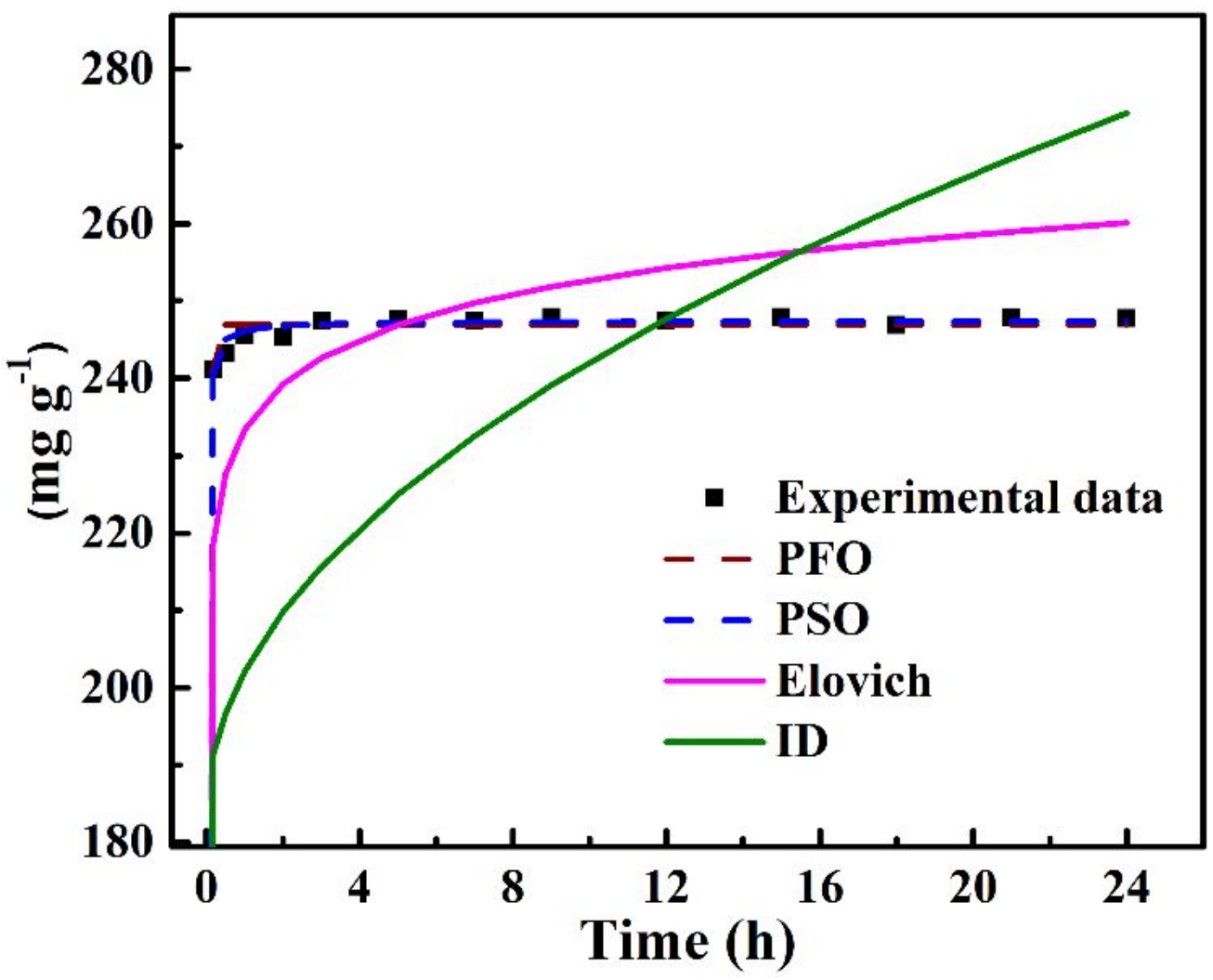

Fig. S5. Fits of the adsorption kinetics models with Pb(II) ions.

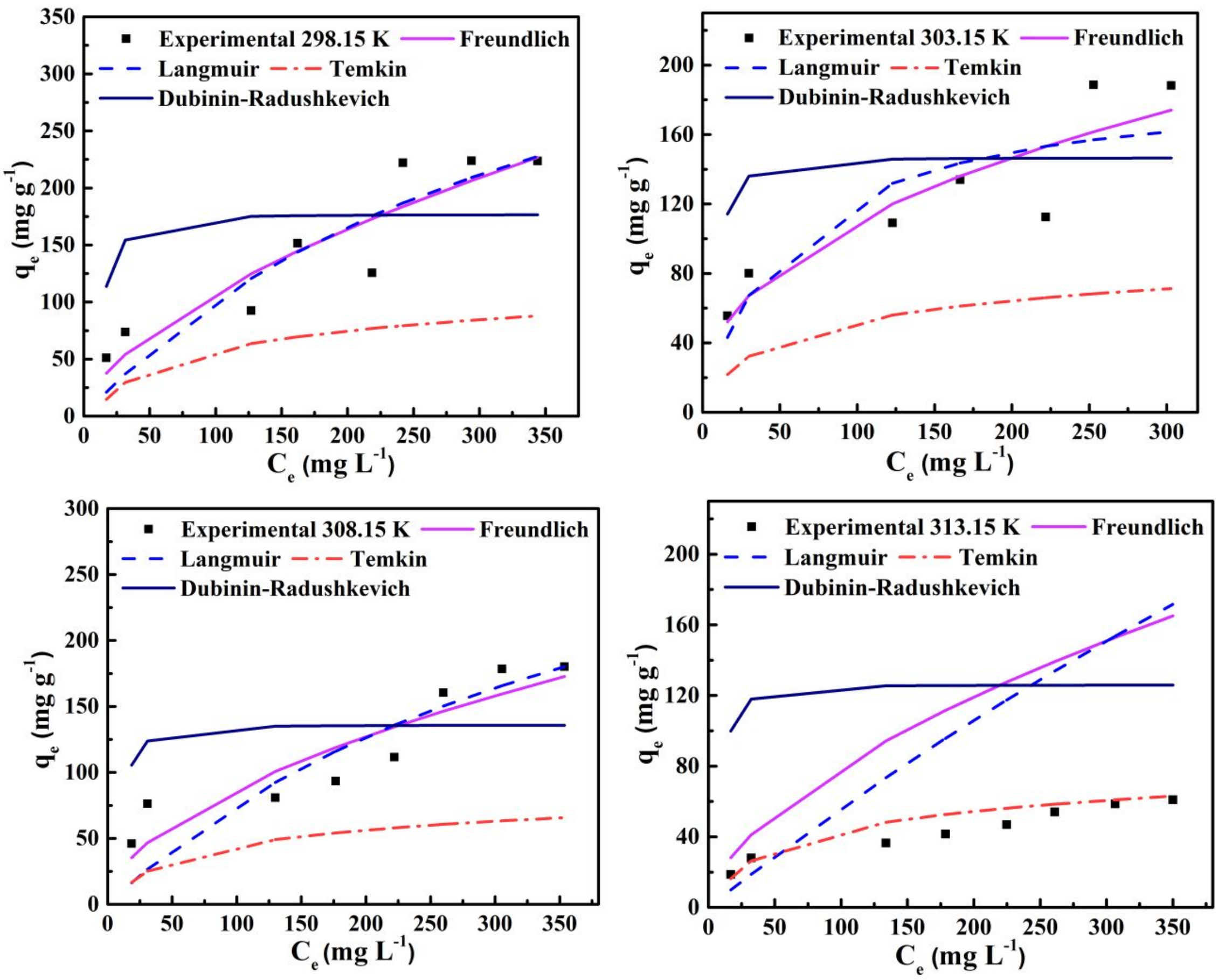

Fig. S6. Results of adjustments of the equilibrium isotherm models of the adsorption of Pb(II) ions with the BMIGA adsorbent, at different temperatures.

Table S5- Adsorption capacity (q_e) and Pb(II) removal efficiency obtained at different adsorbent dosages.

**Adsorbent dosage (g L^{-1})**	**Adsorption capacity, q_e (mg g^{-1})**	**Removal (%)**
0.25	285.86	35.73
0.50	157.13	39.28
1.00	90.67	45.33
1.50	69.76	52.32
2.00	59.14	59.14
2.50	50.55	63.18

Table S6. Results of the parameters of the kinetic models of the adsorption of Pb(II) ions with BMIGA.

**Models**	**Parameters**
PFO	q_1 = 246.90 mg g^{-1}; K_1 = 22.46 h^{-1}; R^2 = 0.9996; χ^2 = 0.0927; RMSE = 1.38
PSO	q_2 = 247.40 mg g^{-1}; K_2 = 0.8165 g mg^{-1} h^{-1}; R^2 = 0.9998; χ^2 = 0.0327; RMSE = 0.81
Elovich	α = 1.02x10^{13} g mg^{-1} h^{-1}; β = 0.1192 mg g^{-1}; R^2 = 0.9714; χ^2 = 3.11x10^{18}; RMSE = 11.14
ID	C_{ID} = 183.6 mg g^{-1}; K_{ID} = 18.51 mg g^{-1} $h^{-0,5}$; R^2 = 0.1410; χ^2 = 52.93; RMSE = 61.07

Table S7. Results of the parameters of the chemical equilibrium models of adsorption of Pb(II) ions with BMIGA.

Models	Parameters			
	298.15 K	303.15 K	308.15 K	313.15 K
Freundlich	q_{mF} = 226.8<br>K_F = 6.87<br>n_F = 1.6701<br>$1/n_F$ = 0.59<br>R^2 = 0.8131<br>χ^2 = 42.90<br>RMSE = 30.75	q_{mF} = 174.0<br>K_F = 16.62<br>n_F = 2.4326<br>$1/n_F$ = 0.41<br>R^2 = 0.7742<br>χ^2 = 20.22<br>RMSE = 24.05	q_{mF} = 172.6<br>K_F = 7.40<br>n_F = 1.8632<br>$1/n_F$ = 0.54<br>R^2 = 0.7971<br>χ^2 = 39.19<br>RMSE = 22.99	q_{mF} = 165.0<br>K_F = 5.46<br>n_F = 1.7184<br>$1/n_F$ = 0.58<br>R^2 = 313.55<br>χ^2 = 4.5x10^3<br>RMSE = 27.59
Langmuir	q_{mL} = 472.7<br>K_L = 0.0027<br>R^2 = 0.7675<br>χ^2 = 108.29<br>RMSE = 34.30	q_{mL} = 191.3<br>K_L = 0.0181<br>R^2 = 0.6649<br>χ^2 = 32.13<br>RMSE = 29.29	q_{mL} = 401.7<br>K_L = 0.0023<br>R^2 = 0.6919<br>χ^2 = 157.68<br>RMSE = 28.32	q_{mL} = 988.7<br>K_L = 0.0006<br>R^2 = 0.6274<br>χ^2 = 281.65<br>RMSE = 30.89
Sips	q_{ms} = 5.1x10^3<br>n_s = 0.4903<br>K_s = 0.6160<br>R^2 = 0.7572<br>χ^2 = 38047.79<br>RMSE = 35.06	q_{ms} = 2.0x10^3<br>n_s = 0.3888<br>K_s = 5.6x10^{-6}<br>R^2 = 0.7155<br>χ^2 = 695.91<br>RMSE = 26.99	q_{ms} = 2.1x10^3<br>n_s = 0.4862<br>K_s = 1.4x10^{-5}<br>R^2 = 0.7512<br>χ^2 = 39.11<br>RMSE = 25.45	q_{ms} = 4.4x10^3<br>n_s = 0.4760<br>K_s = 2.5x10^{-6}<br>R^2 = 0.6293<br>χ^2 = 303.88<br>RMSE = 30.81
Temkin	K_T = 0.1063<br>B = 56.24<br>b_T = 44.07<br>R^2 = 0.7084<br>χ^2 = 995.79<br>RMSE = 38.42	K_T = 0.2267<br>B = 38.81<br>b_T = 64.94<br>R^2 = 07148<br>χ^2 = 696.71<br>RMSE = 27.02	K_T = 0.1465<br>B = 39.34<br>b_T = 65.12<br>R^2 = 0.6709<br>χ^2 = 828.41<br>RMSE = 29.27	K_T = 0.1727<br>B = 35.35<br>b_T = 73.65<br>R^2 = 0.5433<br>χ^2 = 7.63<br>RMSE = 34.20
D-R	q_{DR} = 176.6<br>β = 5.69<br>E = 0.2964<br>R^2 = 0.1675<br>χ^2 = 170.76<br>RMSE = 64.91	q_{DR} = 146.5<br>β = 1.94<br>E = 0.5076<br>R^2 = 0.1387<br>χ^2 = 95.44<br>RMSE = 46.97	q_{DR} = 135.8<br>β = 2.16<br>E = 0.4811<br>R^2 = 0.0921<br>χ^2 = 123.22<br>RMSE = 48.62	q_{DR} = 125.9<br>β = 2.26<br>E = 0.4703<br>R^2 = -0.1554<br>χ^2 = 413.62<br>RMSE = 54.40

Units: q_{mF} mg g^{-1}; k_F (mg g^{-1})(mg^{-1} L^{-1})^{nF}; q_{mL} mg g^{-1}; kL mg L^{-1}; q_{ms} mg g^{-1}; k_T L mol^{-1}; B mg g^{-1}; b_T J mol^{-1}; q_{DR} mg g^{-1}; β mol^2 kJ^{-2}; E kJ mol^{-1}

Table S8. Experimental parameters of the Thomas (TH), Yoon–Nelson (YN), and Clark (CL) models obtained from fixed-bed column adsorption at different initial concentrations and flow rates of Pb(II) ions.

				**Parameters**					
**C_0**	**t_b**	**t_s**	**MTZ**	**TH**	**YN**		**CL**		**q_e**
				**K_{TH}**	**K_{YN}**	**τ**	**r**	**A (x 10^5)**	
400.0	554.88	730.00	1.679	0.5323	0.0320	646.03	0.0320	9272.7	104.94
600.0	341.62	511.41	2.324	0.9947	0.0285	434.10	0.0318	2.3869	126.44
800.0	226.99	370.12	2.706	0.9893	0.0365	306.01	0.0365	0.69892	180.50

Units: C_0 = mg L^{-1}; t_b = min; t_s = min; MTZ = cm; K_{TH} = mL/min mg; K_{YN} = L/min; τ = min; r = min; q_e = mg g^{-1}

REFERENCES

(1) Revellame, E. D.; Fortela, D. L.; Sharp, W.; Hernandez, R.; Zappi, M. E. Adsorption Kinetic Modeling Using Pseudo-First Order and Pseudo-Second Order Rate Laws: A Review. *Clean. Eng. Technol* 2020, *1,* 100032.

(2) Ezzati, R. A New Insight into the Pseudo-Second-Order Model and the Physical Meaning of Its Rate Constant in Adsorption. *J. Disp. Sci. Technol* 2025, *46 (2),* 222–229.

(3) Lin, T. Y.; Xie, K. H.; Wu, C. Y.; Wang, L. Y.; Liu, C. H.; Shu, C. C. Removal of Anionic Dyes from an Aqueous Solution by a Polyaniline-Modified Bioflocculant. J. Taiwan Inst. *Chem. Eng* 2025, *177,* 106302.

(4) Wang, J.; Guo, X. Rethinking of the Intraparticle Diffusion Adsorption Kinetics Model: Interpretation, Solving Methods and Applications. *Chemosphere* 2022, 309

(5) Al-ghouti, M. A.; Da, D. A. Guidelines for the Use and Interpretation of Adsorption Isotherm Models: A Review. *J. Hazard. Mater* 2020, *393,* 122383.

(6) Yaffar, D.; Brenner, J.; Walker, A. P.; Craig, M. E.; Vaughan, E.; Marín-Spiotta, E.; Matos, M.; Rios, S.; Mayes, M. A. The Freundlich Isotherm Equation Best Represents Phosphate Sorption across Soil Orders and Land Use Types in Tropical Soils of Puerto Rico. *Biogeochemistry* 2025, *168 (2),* 1–15.

(7) Lach, J. Analysis of the Differences Resulting from the Determination of Langmuir Isotherm Coefficients from Linear and Non-Linear Forms—A Case Study. *Materials* 2025, *18 (15),* 3506.

(8) Almeida-Naranjo, C. E.; Aldás, M. B.; Cabrera, G.; Guerrero, V. H. Caffeine Removal from Synthetic Wastewater Using Magnetic Fruit Peel Composites:

Material Characterization, Isotherm and Kinetic Studies. *Environ. Chall* 2021, *5,* 100343.

(9) Yusuff, A. S.; Popoola, L. T.; Ibrahim, I. S. Adsorptive Removal of Anthraquinone Dye from Wastewater Using Silica-Nitrogen Reformed Eucalyptus Bark Biochar: Parametric Optimization, Isotherm and Kinetic Studies. J. Taiwan Inst. *Chem. Eng* 2025, *166,* 105503.

(10) Hu, Q.; Zhang, Z. Application of Dubinin–Radushkevich Isotherm Model at the Solid/Solution Interface: A Theoretical Analysis. J. Mol. Liq 2019, 277, 646–648.

(11) Mary Lissy, P. N.; Madhu, G.; Thomas, R. M. Numerical Modelling of a Fixed Bed Column for the Cr(VI) Adsorption Using Pyrrole Coated Nanocomposite. *J. Indian Chem. Soc* 2025, *102 (*2), 101568.

(12) Hu, Q.; Zhang, Y.; Pei, Q.; Li, S. Determination of Equilibrium Loading by Empirical Models for the Modeling of Breakthrough Curves in a Fixed-Bed Column: From Experience to Practice. *Water* 2025, *17*, 329.

(13) Bhagawati, P. B.; Adeogun, A. I.; Shivayogimath, C. B.; Kadier, A. Gum Arabic Tree Biomass Derived Activated Carbon for Fluoride Sequestration in Batch and Fixed Bed Processes: Kinetics, Thermodynamics and Column Adsorption Modeling. *J. Disp. Sci. Technol* 2025, *46 (4),* 619–627.

(14) Aniagor, C. O.; Menkiti, M. C. Application of Modified Yoon-Nelson Nonlinear Equation for Modeling Antibiotics Adsorption onto a Composite Adsorbent. Results Surf. *Interfaces* 2025, *18*, 100370.

(15) Gerc, H. F. Adsorption of Lead(II) Ions from Aqueous Solutions by Activated Carbon Prepared from Biomass Plant Material of Euphorbia Rigida. *Chem. Eng. J* 2007, *132*, 289–297.

(16) Pehlivan, E.; Altun, T.; Cetin, S.; Bhanger, M. I. Lead Sorption by Waste Biomass of Hazelnut and Almond Shell. *J. Hazard. Mater* 2009, *167,* 1203–1208.

(17) Swaminaathan, P.; Kamalesh, R.; Karishma, S.; Ragini, Y. P.; Saravanan, A.; Vickram, A. S. A Comparative Study of Raw and Sulfuric Acid-Activated Areca-Fungal Biochar for Effective Pb (II) Adsorption. *Next Materials* 2026, *10*, 101360.

(18) Ashfaq, A.; Nadeem, R.; Bibi, S.; Rashid, U.; Hanif, M. A.; Jahan, N.; Ashfaq, Z.; Ahmed, Z.; Adil, M.; Naz, M. Efficient Adsorption of Lead Ions from Synthetic Wastewater Using Agrowaste-Based Mixed Biomass *Water* 2021, *13*, 3344.

(19) Radenkovi, M.; Petrovi, J. Waste Biomass Derived Highly-Porous Carbon Material for Toxic Metal Removal: Optimisation, Mechanisms and Environmental Implications. *Chemosphere* 2024, *347*, 140684.

(20) El, A.; Rachid, A.; Assia, H.; Iman, J.; Jaouad, L.; Fatima, B.; Bouhassane, Z. Acid Activation of Natural Reed Filter Biomass (*Typha Latifolia*) Application to Pb(II) Uptake from Aqueous Solutions: Kinetic, Thermodynamic Equilibrium Studies and Optimization Studies. *Chem. Afr.* 2024, *7*, 345–365.

(21) Fodil, M.; Maane, S.; Avalos Ramirez, A.; Delgado Cano, B.; Haroun, M. F. Adsorption of Lead from Wastewater Using Olive Leaf Powder as Biosorbent. *Int. J. Environ. Sci. Technol.* 2024, *21*, 2615–2626

(22) Özer, Ç.; İmamoğlu, M. Isolation of Nickel(II) and Lead(II) from Aqueous Solution by Sulfuric Acid Prepared Pumpkin Peel Biochar. *Anal. Lett* 2023, 491-503

(23) Mine, Z.; Zehra, Ş.; Keskin, S.; Dinçer, E.; Ben, A. Influential Lead Uptake Using Dried and Inactivated ‑ Fungal Biomass Obtained from Panaeolus Papilionaceus : Biological Activity, Equilibrium, and Mechanism. *Biomass Convers. Biorefin* 2024, *15*, 7283–7294
